# Plasma fatty acid levels and risk of non-small cell lung cancer: a large-scale prospective cohort study

**DOI:** 10.3389/fnut.2024.1462300

**Published:** 2024-09-18

**Authors:** Hua-Long Lin, Qiu-Yan Lin, Jie-Ni Feng, Wei-E Zheng, Chuang Yang, Shao-Fei Yuan

**Affiliations:** ^1^Department of Medical Oncology, Rui'an People's Hospital, The Third Affiliated Hospital of Wenzhou Medical University, Wenzhou, China; ^2^Department of Chemoradiation and Oncology, The Third Affiliated Hospital of Wenzhou Medical University, Wenzhou, China; ^3^Medical Faculty, University of Leipzig, Leipzig, Germany

**Keywords:** non-small cell lung cancer, fatty acids, UK Biobank, prospective cohort study, cancer prevention

## Abstract

**Background:**

Non-small cell lung cancer (NSCLC) ranks among the most prevalent and lethal malignancies globally. Fatty acids (FAs) play a significant role in diverse physiological and pathological mechanisms, yet their precise involvement in NSCLC remains poorly understood.

**Methods:**

This study utilized a large-scale prospective cohort of 249,132 participants, observed over an average of 12 years, to investigate the relationship between different FAs and NSCLC risk. Analytical approaches included Cox proportional hazards regression, Kaplan–Meier survival analysis, accelerated failure time (AFT) modeling, and restricted cubic spline (RCS) analysis.

**Results:**

During the follow-up period, 1,460 participants were diagnosed with NSCLC. Cox regression analysis demonstrated that elevated levels of docosahexaenoic acid (DHA), linoleic acid (LA), and omega-3 were inversely associated with NSCLC risk. Kaplan–Meier curves, along with AFT models, corroborated that elevated concentrations of DHA and LA significantly delayed NSCLC onset. Additionally, RCS analysis uncovered nuanced dose–response relationships between these FAs and NSCLC. Stratified analyses highlighted variability based on smoking status, gender, and body mass index subgroups.

**Conclusion:**

The concentration of specific FAs exhibits a significant association with NSCLC risk. These results offer a foundation for devising dietary FA composition adjustments aimed at reducing NSCLC risk.

## Introduction

Non-small cell lung cancer (NSCLC), accounting for approximately 85% of lung cancer cases, stands as one of the most prevalent and lethal malignancies globally. Lung cancer represents nearly 12.4% of all cancer incidences and is responsible for 18.7% of cancer-related mortality worldwide. In 2022 alone, the global burden of lung cancer was marked by nearly 2.5 million new cases and over 1.8 million deaths (1, 2). Despite ongoing advancements in medical technology, early detection and effective treatment of NSCLC remain formidable challenges, contributing to persistently low five-year survival rates (3). The high prevalence and mortality associated with NSCLC highlight the necessity of investigating its etiology and risk factors, which are essential for developing effective preventive and therapeutic strategies (4).

Fatty acids (FAs), prevalent biomolecules in the human body, are integral to cell membrane composition and participate in various physiological and pathological mechanisms (5). The link between FAs and cancer has garnered heightened research interest, particularly concerning their role in oncogenesis. Among FAs, omega-3 and omega-6 polyunsaturated varieties are hypothesized to significantly influence cancer development and progression (6, 7). While omega-3 FAs are associated with anti-inflammatory and antitumorigenic effects, omega-6 FAs are implicated in promoting inflammation and tumorigenesis (8). Nevertheless, the relationship between specific FA types and NSCLC risk remains ambiguous, with studies yielding incongruent outcomes (9, 10). Consequently, further investigation into FAs and NSCLC risk is imperative to inform novel strategies for NSCLC prevention and treatment.

This study utilized data from a large-scale prospective cohort to investigate the association between FA levels and NSCLC risk. It aimed to generate scientific insights that could inform strategies for NSCLC prevention and early intervention while enhancing comprehension of the potential oncological implications of FAs.

## Methods

### Study population

The UK Biobank (UKB) represents an extensive prospective cohort study designed to rigorously examine genetic and environmental factors influencing disease prevalence in middle-aged and elderly populations. Conducted between 2006 and 2010, the study enrolled more than 500,000 individuals aged 37–73 years, systematically collecting comprehensive baseline data and biological samples. All participants voluntarily provided written informed consent, with ethical clearance obtained from the North West Multicenter Research Ethics Committee. Study details have been elaborated in prior publications (11).

### Measurement of FAs

Metabolomic profiling of baseline plasma samples from over 270,000 UK Biobank participants was performed by Nightingale Health Laboratories utilizing a high-throughput nuclear magnetic resonance (NMR) metabolomics platform (12, 13). The initial measurements were carried out between June 2019 and April 2020 (Phase 1), followed by a second round between April 2020 and June 2022 (Phase 2). The analysis included 251 biological metabolites such as lipoproteins, FAs, amino acids, among others. Comprehensive details on the NMR protocols are available on the UK Biobank website.^1^ This study specifically focused on 17 FAs from the NMR project, encompassing total FAs, docosahexaenoic acid (DHA), DHA/FA, linoleic acid (LA), LA/FA, monounsaturated fatty acids (MUFA), MUFA/FA, omega-3 FAs (omega-3), omega-3/FA, omega-6 FAs (omega-6), omega-6/omega-3, omega-6/FA, polyunsaturated fatty acids (PUFA), PUFA/MUFA, PUFA/FA, saturated fatty acids (SFA), and SFA/FA.

### Assessment of outcome

NSCLC diagnoses were identified using International Classification of Diseases, Tenth Revision (ICD-10) code C34, as documented in the cancer registry records at UKB (Supplementary Table S1). The follow-up commenced from the participants’ enrollment date and extended to the earliest of the following events: first NSCLC diagnosis, occurrence of other cancers, death, or the study’s cutoff date (June 01, 2022).

### Assessment of other covariates

Demographic and medical data of participants were comprehensively gathered at baseline via interviews or online questionnaires. Collected variables included age, sex, ethnicity, BMI, physical activity, fasting duration, dietary patterns, Townsend Deprivation Index (TDI), smoking and alcohol consumption, cardiovascular disease (CVD) and diabetes mellitus (DM) history, and familial cancer history. Data on lipid-lowering and insulin use were also documented. BMI was computed as weight (kg) divided by height squared (m^2^). Physical activity was quantified through metabolic equivalent task (MET) minutes (14), while TDI served as an indicator of socioeconomic status (15). Dietary patterns were scored from 0 to 9 based on the intake of nine specific food categories, with higher scores reflecting a higher intake of unhealthy food; detailed criteria for this scoring system were available elsewhere (16). Familial cancer history was defined as a parental history of cancer diagnosis.

### Selection criteria

Initial data collection included 502,357 participants. Exclusions were made for 45,777 individuals with a prior cancer diagnosis and two individuals due to missing recruitment time records. An additional 207,446 participants were excluded due to incomplete FA data. Ultimately, the study cohort comprised 249,132 participants.

### Statistical analysis

Baseline covariates with missing data were imputed using random forest multiple imputation, generating five datasets. For further analysis, one dataset was randomly chosen. Baseline characteristics were then stratified by NSCLC onset. Continuous variables were summarized as medians with interquartile ranges, while categorical variables were presented as frequencies and proportions (*N*, %). Group comparisons employed the Kruskal–Wallis test for continuous variables and the chi-square test for categorical variables.

The FA data underwent z-score normalization before analysis. Cox proportional hazard models were utilized to estimate hazard ratios (HRs) and 95% confidence intervals (CIs), examining the relationship between each FA’s standard deviation (SD) and NSCLC risk. Three multivariate Cox regression models were developed: Model 1 served as the unadjusted baseline; Model 2 included adjustments for age, sex, and race; while Model 3 incorporated additional adjustments for BMI, MET, TDI, smoking and alcohol consumption, diet score, fasting duration, cancer family history, lipid-lowering medications, insulin use, and chronic disease history, including DM and CVD.

Subsequently, FAs were stratified into quartiles, followed by the application of Kaplan–Meier analysis to assess NSCLC incidence across these quartiles, with significance determined via the log-rank test. To explore the exposure–response relationship between FAs and NSCLC, restricted cubic spline (RCS) analysis was performed, utilizing three knots positioned at the 10th, 50th, and 90th percentiles, and non-linear *p*-values were derived from the likelihood ratio test (17). An accelerated failure time (AFT) model, incorporating the Weibull distribution, was employed to evaluate the influence of FA levels on the latency period to NSCLC onset (18). This analysis adjusted for multiple covariates, using the lowest FA quartile (Q1) as the reference point to assess the impact of increasing quartiles on NSCLC onset timing. Negative coefficients corresponded to a delayed NSCLC onset, while positive coefficients indicated an accelerated onset.

In addition, subgroup analyses were performed according to sex, age, race, BMI, smoking and drinking habits, family history of cancer, DM, CVD, and lipid-lowering drug usage to determine the effect of SD increases in each FA on NSCLC risk across different cohorts. Interaction *p*-values were derived via likelihood ratio tests. To ensure the robustness of the results, multiple sensitivity analyses were executed. Initially, participants with follow-up periods shorter than 2 years were excluded to mitigate the influence of reverse causality. Subsequently, cases with missing baseline covariate values were omitted to evaluate the effect of imputation on the outcomes. Lastly, consistency of the results was confirmed using four additional datasets with multiple imputations. All analyses were conducted using R software (version 4.3.1), with statistical significance defined as a two-sided *p*-value <0.05.

## Results

### Baseline characteristics

The analysis cohort comprised 249,132 participants, among whom 1,460 were diagnosed with NSCLC during a mean follow-up of 12.0 years (Table 1). Compared to the non-NSCLC group (*n* = 247,672), individuals with NSCLC were generally older (median age 62 vs. 57 years, *p* < 0.001), more likely to be male (51.4% vs. 47.1%, *p* < 0.001), and predominantly White (96.5% vs. 94.5%, *p* < 0.001). Furthermore, the NSCLC group presented with lower metabolic values, elevated TDI and dietary scores, and higher prevalences of diabetes and cardiovascular diseases. Notable differences were also identified in FA levels and ratios between the NSCLC and non-NSCLC cohorts (Table 1).

**Table 1:** Baseline demographic and clinical characteristics.

Characteristic	Total	Non-NSCLC	NSCLC	*P*-value
	(*n* = 249,132)	(*n* = 247,672)	(*n* = 1,460)	
Age, years	57.0 (50.0–63.0)	57.0 (50.0–63.0)	62.0 (58.0–66.0)	<0.001
Male, *N* (%)	117,386 (47.1%)	116,636 (47.1%)	750 (51.4%)	<0.001
White, *N* (%)	23,5,339 (94.5%)	233,930 (94.5%)	1,409 (96.5%)	<0.001
MET	1789.0 (813.0–3573.0)	1790.0 (813.0–3573.0)	1711.5 (678.0–3546.0)	0.022
Townsend deprivation index	−2.2 (−3.7–0.5)	−2.2 (−3.7–0.5)	−0.8 (−3.1–2.6)	<0.001
BMI (kg/m^2^)	26.8 (24.2–29.9)	26.8 (24.2–29.9)	26.8 (24.2–30.0)	0.758
Diet score	5.0 (4.0–6.0)	5.0 (4.0–6.0)	6.0 (4.0–7.0)	<0.001
DM, *N* (%)	13,063 (5.2%)	12,929 (5.2%)	134 (9.2%)	<0.001
CVD, *N* (%)	19,714 (7.9%)	19,476 (7.9%)	238 (16.3%)	<0.001
Lipid-lowering drugs, *N* (%)	43,761 (17.6%)	43,314 (17.5%)	447 (30.6%)	<0.001
Insulin, *N* (%)	2,767 (1.1%)	2,744 (1.1%)	23 (1.6%)	0.089
History of cancer family	74,362 (29.8%)	73,847 (29.8%)	515 (35.3%)	
Drinking status, *N* (%)				<0.001
Never	10,884 (4.4%)	10,834 (4.4%)	50 (3.4%)	
Previous	8,801 (3.5%)	8,701 (3.5%)	100 (6.8%)	
Current	229,447 (92.1%)	228,137 (92.1%)	1,310 (89.7%)	
Smoking status, *N* (%)				<0.001
Never	100,633 (40.4%)	100,507 (40.6%)	126 (8.6%)	
Previous	122,272 (49.1%)	121,531 (49.1%)	741 (50.8%)	
Current	26,227 (10.5%)	25,634 (10.3%)	593 (40.6%)	
DHA (mmol/l)	0.2 (0.2–0.3)	0.2 (0.2–0.3)	0.2 (0.2–0.3)	<0.001
DHA/FA	1.9 (1.5–2.3)	1.9 (1.5–2.3)	1.8 (1.4–2.2)	<0.001
LA (mmol/l)	3.4 (3.0–3.9)	3.4 (3.0–3.9)	3.3 (2.8–3.8)	<0.001
LA/FA	29.1 (26.8–31.3)	29.1 (26.8–31.3)	27.6 (25.2–29.9)	<0.001
MUFA (mmol/l)	2.8 (2.3–3.3)	2.8 (2.3–3.3)	2.9 (2.4–3.5)	<0.001
MUFA/FA	23.5 (21.8–25.4)	23.5 (21.8–25.4)	24.3 (22.6–26.4)	<0.001
Omega.3 (mmol/l)	0.5 (0.4–0.6)	0.5 (0.4–0.6)	0.5 (0.4–0.6)	0.291
Omega.3/FA	4.2 (3.3–5.1)	4.2 (3.3–5.1)	4.0 (3.2–4.9)	0.004
Omega.6 (mmol/l)	4.5 (4.0–4.9)	4.5 (4.0–4.9)	4.3 (3.9–4.9)	<0.001
Omega.6/Omega.3	9.1 (7.2–11.5)	9.1 (7.2–11.5)	9.2 (7.3–11.8)	0.039
Omega.6/FA	38.4 (35.7–40.5)	38.4 (35.8–40.5)	37.3 (34.4–39.5)	<0.001
PUFA (mmol/l)	5.0 (4.5–5.5)	5.0 (4.5–5.5)	4.8 (4.3–5.4)	<0.001
PUFA/MUFA	1.8 (1.6–2.1)	1.8 (1.6–2.1)	1.7 (1.5–1.9)	<0.001
PUFA/FA	42.8 (40.1–44.9)	42.8 (40.1–44.9)	41.5 (38.6–43.6)	<0.001
SFA (mmol/l)	4.0 (3.4–4.6)	4.0 (3.4–4.6)	4.1 (3.5–4.8)	<0.001
SFA/FA	33.9 (32.7–35.1)	33.9 (32.7–35.1)	34.3 (33.1–35.7)	<0.001
FA (mmol/l)	11.8 (10.4–13.4)	11.8 (10.4–13.4)	11.8 (10.4–13.7)	0.274

NSCLC, non-small cell lung cancer; MET, metabolic equivalents; BMI, body mass index; DM, diabetes mellitus; CVD, cardiovascular disease; DHA, docosahexaenoic acid; DHA/FA, docosahexaenoic acid to total fatty acids percentage; LA, linoleic acid; LA/FA, linoleic acid to total fatty acids percentage; MUFA, monounsaturated fatty acids; MUFA/FA, monounsaturated fatty acids to total fatty acids percentage; Omega-3, Omega-3 fatty acids; Omega-3/FA, Omega-3 fatty acids to total fatty acids percentage; Omega-6, Omega-6 fatty acids; Omega-6/Omega-3, Omega-6 fatty acids to Omega-3 fatty acids ratio; Omega-6/FA, Omega-6 fatty acids to total fatty acids percentage; PUFA, polyunsaturated fatty acids; PUFA/MUFA, polyunsaturated fatty acids to monounsaturated fatty acids ratio; PUFA/FA, polyunsaturated fatty acids to total fatty acids percentage; SFA, saturated fatty acids; SFA/FA, saturated fatty acids to total fatty acids percentage; FA, total fatty acids.

### FAs and their association with NSCLC risk

Cox regression analysis identified significant correlations between specific FAs and their ratios with NSCLC risk. Across all three models, even after adjustments, DHA, LA, omega-3, and PUFA were consistently linked to a lower NSCLC risk (Table 2). Notably, DHA exhibited a pronounced protective effect (HR = 0.89, 95% CI: 0.84–0.95, *p* < 0.001). In contrast, both MUFA and SFA were significantly associated with an elevated NSCLC risk, with MUFA showing the highest risk in unadjusted models (HR = 1.14, 95% CI: 1.09–1.20, *p* < 0.001). This association, however, weakened after comprehensive adjustment (HR = 1.02, 95% CI: 0.97–1.07, *p* = 0.462) (Table 2). Analysis of FA ratios revealed that elevated DHA/FA, LA/FA, omega-3/FA, and PUFA/FA ratios correlate with reduced NSCLC risk, while increased MUFA/FA and SFA/FA ratios correlate with heightened risk (e.g., SFA/FA HR = 1.12, 95% CI: 1.06–1.17, *p* < 0.001 after full adjustment) (Table 2). This evidence emphasizes the significant influence of FAs on NSCLC onset, suggesting their potential in prevention.

**Table 2:** The association between circulating fatty acids and the risk of NSCLC.

Type	Model 1		Model 2		Model 3	
	HR (95% CI)	*P*	HR (95% CI)	*P*	HR (95% CI)	*P*
DHA	0.83 (0.79–0.88)	<0.001	0.75 (0.7–0.79)	<0.001	0.89 (0.84–0.95)	<0.001
DHA/FA	0.81 (0.77–0.86)	<0.001	0.76 (0.71–0.8)	<0.001	0.9 (0.85–0.96)	<0.001
LA	0.8 (0.76–0.85)	<0.001	0.82 (0.78–0.87)	<0.001	0.88 (0.83–0.93)	<0.001
LA/FA	0.68 (0.65–0.71)	<0.001	0.75 (0.71–0.79)	<0.001	0.84 (0.79–0.89)	<0.001
MUFA	1.14 (1.09–1.2)	<0.001	1.1 (1.05–1.16)	<0.001	1.02 (0.97–1.07)	0.462
MUFA/FA	1.36 (1.29–1.42)	<0.001	1.32 (1.26–1.39)	<0.001	1.12 (1.06–1.18)	<0.001
Omega-3	0.9 (0.85–0.95)	<0.001	0.79 (0.74–0.83)	<0.001	0.91 (0.86–0.96)	<0.001
Omega-3/FA	0.87 (0.82–0.91)	<0.001	0.76 (0.71–0.8)	<0.001	0.9 (0.84–0.95)	<0.001
Omega-6	0.85 (0.8–0.89)	<0.001	0.85 (0.8–0.89)	<0.001	0.9 (0.85–0.95)	<0.001
Omega-6/Omega-3	1.07 (1.03–1.11)	<0.001	1.1 (1.08–1.12)	<0.001	1.07 (1.04–1.11)	<0.001
Omega-6/FA	0.75 (0.71–0.78)	<0.001	0.81 (0.77–0.85)	<0.001	0.9 (0.86–0.95)	<0.001
PUFA	0.84 (0.8–0.89)	<0.001	0.81 (0.77–0.86)	<0.001	0.89 (0.84–0.94)	<0.001
PUFA/MUFA	0.7 (0.66–0.74)	<0.001	0.73 (0.69–0.77)	<0.001	0.86 (0.81–0.91)	<0.001
PUFA/FA	0.72 (0.69–0.76)	<0.001	0.75 (0.71–0.78)	<0.001	0.87 (0.82–0.91)	<0.001
SFA	1.09 (1.03–1.14)	<0.001	1.04 (0.99–1.1)	0.121	1 (0.95–1.06)	0.881
SFA/FA	1.27 (1.21–1.34)	<0.001	1.23 (1.17–1.29)	<0.001	1.12 (1.06–1.17)	<0.001
FA	1.03 (0.98–1.08)	0.24	0.99 (0.94–1.04)	0.631	0.97 (0.92–1.03)	0.319

Model 1: unadjusted; Model 2: adjusted for age, sex, and race; Model 3: adjusted for BMI, MET, TDI, smoking and drinking status, diet score, fasting time, family history of cancer, lipid-lowering drugs, insulin, and history of chronic diseases including DM and CVD. DHA, docosahexaenoic acid; DHA/FA, docosahexaenoic acid to total fatty acids percentage; LA, linoleic acid; LA/FA, linoleic acid to total fatty acids percentage; MUFA, monounsaturated fatty acids; MUFA/FA, monounsaturated fatty acids to total fatty acids percentage; Omega-3, Omega-3 fatty acids; Omega-3/FA, Omega-3 fatty acids to total fatty acids percentage; Omega-6, Omega-6 fatty acids; Omega-6/Omega-3, Omega-6 fatty acids to Omega-3 fatty acids ratio; Omega-6/FA, Omega-6 fatty acids to total fatty acids percentage; PUFA, polyunsaturated fatty acids; PUFA/MUFA, polyunsaturated fatty acids to monounsaturated fatty acids ratio; PUFA/FA, polyunsaturated fatty acids to total fatty acids percentage; SFA, saturated fatty acids; SFA/FA, saturated fatty acids to total fatty acids percentage; FA, total fatty acids.

### Association between FAs and their ratios with time to NSCLC onset

**Figure 1:**
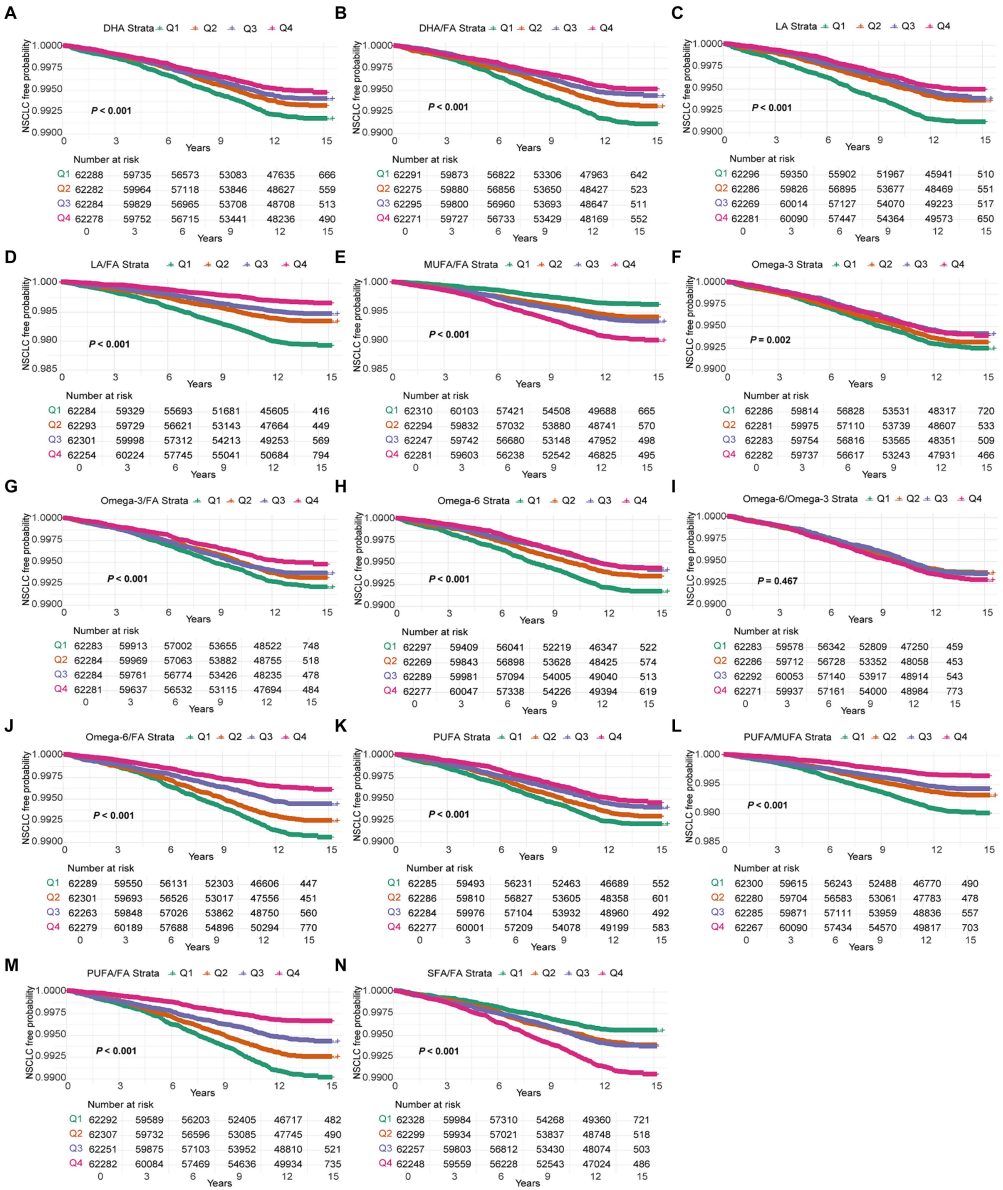
Kaplan–Meier curves for NSCLC events in the 14 fatty acids group. Models were adjusted with age, sex, race, BMI (body mass index), MET (metabolic equivalent task), TDI (Townsend Deprivation Index), smoking and drinking status, diet score, fasting time, family history of cancer, lipid-lowering drugs, insulin, and history of chronic diseases including DM (diabetes mellitus) and CVD (cardiovascular disease). DHA, docosahexaenoic acid; DHA/FA, docosahexaenoic acid to total fatty acids percentage; LA, linoleic acid; LA/FA, linoleic acid to total fatty acids percentage; MUFA/FA, monounsaturated fatty acids to total fatty acids percentage; Omega-3, Omega-3 fatty acids; Omega-3/FA, Omega-3 fatty acids to total fatty acids percentage; Omega-6, Omega-6 fatty acids; Omega-6/Omega-3, Omega-6 fatty acids to Omega-3 fatty acids ratio; Omega-6/FA, Omega-6 fatty acids to total fatty acids percentage; PUFA, polyunsaturated fatty acids; PUFA/MUFA, Polyunsaturated fatty acids to monounsaturated fatty acids ratio; PUFA/FA, polyunsaturated fatty acids to total fatty acids percentage; SFA/FA, saturated fatty acids to total fatty acids percentage; FA, total fatty acids.

Kaplan–Meier survival analysis revealed a significant association between various FAs and their ratios with the time to NSCLC onset. DHA, LA, omega-3, and PUFA, along with their respective ratios (DHA/FA, LA/FA, omega-3/FA, and PUFA/FA), were strongly correlated with a delayed NSCLC onset. Participants in the highest quartile (Q4) exhibited a markedly lower incidence of NSCLC throughout the follow-up period compared to those in quartiles Q1–Q3, with statistically significant differences (*p* < 0.001) (Figure 1). This suggests a substantial protective role of elevated levels of these FAs against NSCLC. In contrast, higher MUFA/FA and SFA/FA ratios were linked to an increased NSCLC risk, with the Q4 group showing a higher incidence early in the follow-up period (*p* < 0.001) (Figure 1). Kaplan–Meier curve results aligned with Cox regression analysis, reinforcing the protective effect of elevated DHA, LA, omega-3, and PUFA levels in NSCLC onset, while highlighting the potential risk linked to higher MUFA/FA and SFA/FA ratios. The FA profile’s significance in formulating NSCLC prevention strategies is thus emphasized.

### Complex dose–response relationships between FAs and NSCLC

**Figure 2:**
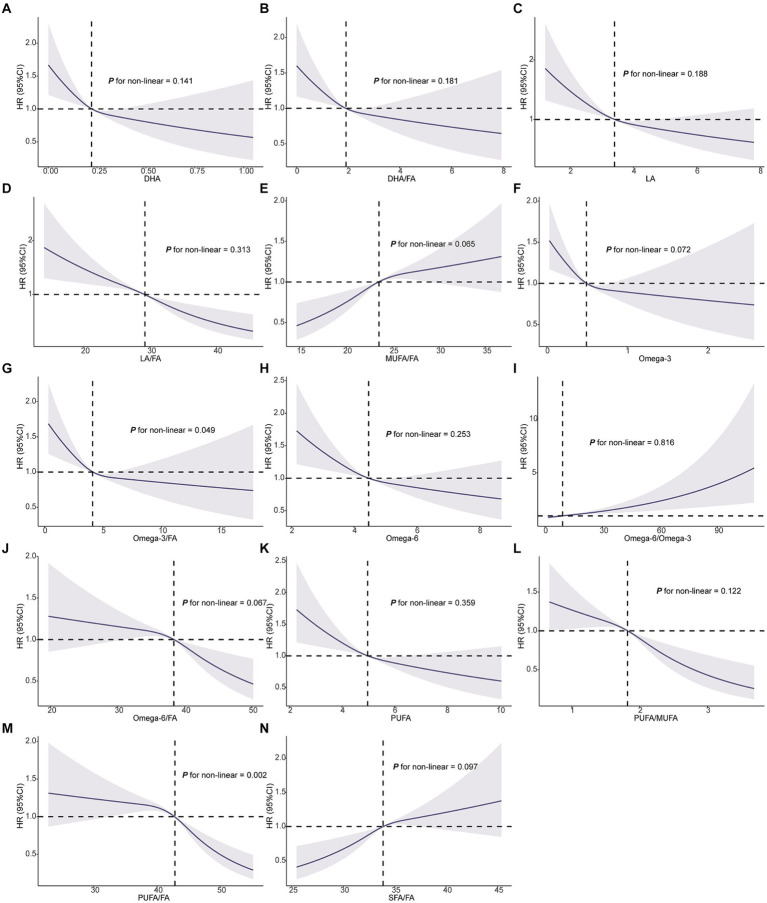
Association of the 14 fatty acids with NSCLC using RCS. Models were adjusted with age, sex, race, BMI (body mass index), MET (metabolic equivalent task), TDI (Townsend Deprivation Index), smoking and drinking status, diet score, fasting time, family history of cancer, lipid-lowering drugs, insulin, and history of chronic diseases including DM (diabetes mellitus) and CVD (cardiovascular disease). DHA, docosahexaenoic acid; DHA/FA, docosahexaenoic acid to total fatty acids percentage; LA, linoleic acid; LA/FA, linoleic acid to total fatty acids percentage; MUFA/FA, monounsaturated fatty acids to total fatty acids percentage; Omega-3, Omega-3 fatty acids; Omega-3/FA, Omega-3 fatty acids to total fatty acids percentage; Omega-6, Omega-6 fatty acids; Omega-6/Omega-3, Omega-6 fatty acids to Omega-3 fatty acids ratio; Omega-6/FA, Omega-6 fatty acids to total fatty acids percentage; PUFA, polyunsaturated fatty acids; PUFA/MUFA, polyunsaturated fatty acids to monounsaturated fatty acids ratio; PUFA/FA, polyunsaturated fatty acids to total fatty acids percentage; SFA/FA, saturated fatty acids to total fatty acids percentage; FA, total fatty acids.

The RCS analysis indicated that most FAs, including DHA, DHA/FA, LA, LA/FA, MUFA/FA, omega-3, omega-6, omega-6/omega-3, omega-6/FA, PUFA, SFA, and SFA/FA, did not demonstrate significant non-linear associations with NSCLC risk (P for non-linearity >0.05) (Figure 2). NSCLC risk predominantly followed a linear pattern with increasing levels of these FAs. In contrast, omega-3/FA (*P* for non-linearity = 0.049) and PUFA/FA (*P* for non-linearity = 0.002) exhibited significant non-linear relationships (Figure 2). Specifically, a lower omega-3/FA ratio correlated with an elevated NSCLC risk, characterized by a steep decline in risk until reaching a threshold, beyond which further increases in the omega-3/FA ratio yielded minimal additional risk reduction. In contrast, the association between PUFA/FA ratios and NSCLC risk revealed only slight variations in risk at lower PUFA/FA levels. However, a marked decrease in risk was observed as PUFA/FA levels increased. In conclusion, RCS analysis further substantiated the intricate relationships between FAs and NSCLC risk, emphasizing the distinct non-linear impact of omega-3/FA and PUFA/FA on risk modulation.

### Impact of FAs on time to NSCLC incidence

**Figure 3:**
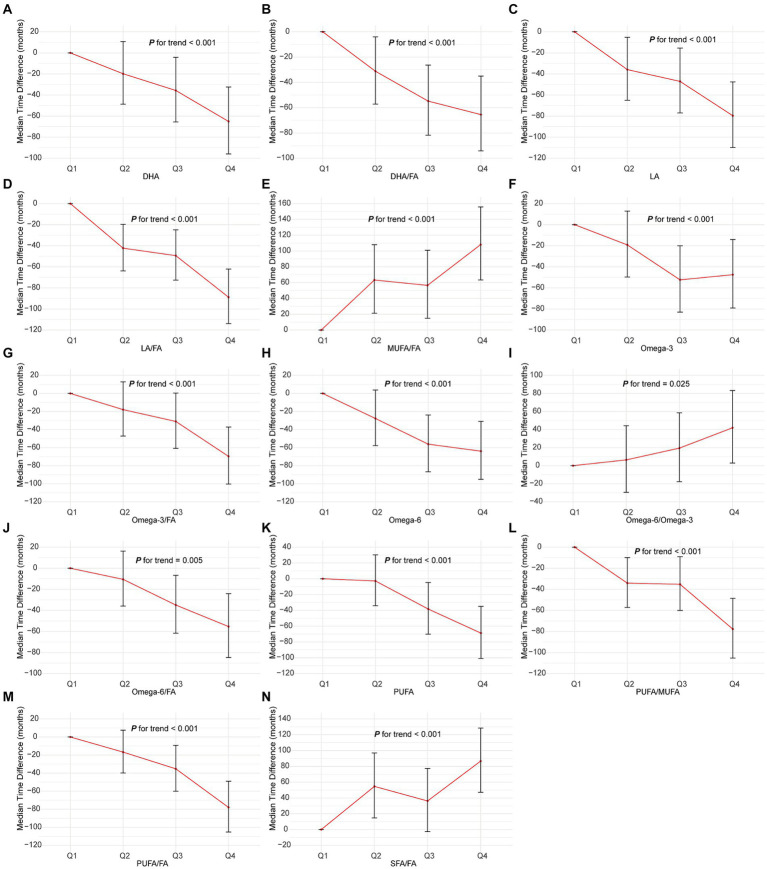
Association of the 14 fatty acids with NSCLC using AFT. Models were adjusted with age, sex, race, BMI (body mass index), MET (metabolic equivalent task), TDI (Townsend Deprivation Index), smoking and drinking status, diet score, fasting time, family history of cancer, lipid-lowering drugs, insulin, and history of chronic diseases including DM (diabetes mellitus) and CVD (cardiovascular disease). DHA, docosahexaenoic acid; DHA/FA, docosahexaenoic acid to total fatty acids percentage; LA, linoleic acid; LA/FA, linoleic acid to total fatty acids percentage; MUFA/FA, monounsaturated fatty acids to total fatty acids percentage; Omega-3, Omega-3 fatty acids; Omega-3/FA, Omega-3 fatty acids to total fatty acids percentage; Omega-6, Omega-6 fatty acids; Omega-6/Omega-3, Omega-6 fatty acids to Omega-3 fatty acids ratio; Omega-6/FA, Omega-6 fatty acids to total fatty acids percentage; PUFA, polyunsaturated fatty acids; PUFA/MUFA, polyunsaturated fatty acids to monounsaturated fatty acids ratio; PUFA/FA, polyunsaturated fatty acids to total fatty acids percentage; SFA/FA, saturated fatty acids to total fatty acids percentage; FA, total fatty acid.

The AFT model analysis revealed that elevated levels of DHA, LA, omega-3, PUFA, and their respective FA ratios (DHA/FA, LA/FA, omega-3/FA, PUFA/FA) were associated with a significant extension in the latency period to NSCLC onset (Figure 3 and Supplementary Table S2). Specifically, the highest quartile (Q4) of DHA corresponded to a delayed onset of NSCLC by 65.06 months (95% CI: 32.31–95.95, *P* for trend <0.001) compared to the lowest quartile (Q1). Similarly, LA in Q4 delayed onset by 79.62 months (95% CI: 47.60–109.83, *P* for trend <0.001), and LA/FA in Q4 by 88.86 months (95% CI: 62.12–113.94, *P* for trend <0.001). Elevated levels of MUFA/FA, SFA/FA, and omega-6/FA were associated with a significantly accelerated onset of NSCLC. Specifically, the Q4 group for MUFA/FA advanced NSCLC onset by 108.15 months (95% CI: 63.45–155.77, *P* for trend <0.001), SFA/FA by 86.66 months (95% CI: 47.15–128.45, *P* for trend <0.001), and omega-6/FA by 41.95 months (95% CI: 2.92–83.22, *P* for trend <0.001). These results highlight the protective role of higher DHA, LA, omega-3, and PUFA levels in delaying NSCLC onset, while elevated MUFA/FA, SFA/FA, and omega-6/FA levels markedly increase the risk by advancing the disease onset.

### Validation of Cox regression results through sensitivity analysis

To ensure the reliability of the Cox regression analysis outcomes, three sensitivity analyses were performed. When excluding NSCLC cases diagnosed within 2 years, both DHA (HR = 0.90, 95% CI: 0.85–0.96, *p* = 0.002) and omega-3 (HR = 0.92, 95% CI: 0.86–0.97, *p* = 0.005) remained associated with a lower NSCLC risk (Supplementary Table S3). Moreover, after excluding participants with incomplete baseline covariate data and utilizing multiple imputations, MUFA/FA (HR = 1.15, 95% CI: 1.07–1.22, *p* < 0.001) and SFA/FA (HR = 1.13, 95% CI: 1.06–1.20, *p* < 0.001) were linked to an elevated NSCLC risk (Supplementary Tables S4, S5). The consistency between these sensitivity analyses and the primary Cox regression results reinforces the significant role of FAs and their ratios in NSCLC risk, thereby enhancing the robustness of the study’s conclusions.

### Influence of FAs on NSCLC risk across different subgroups

Stratified Cox regression analysis was employed to examine the impact of FAs and their ratios on NSCLC risk across different subgroups. The analysis revealed consistent trends in the effects of most FAs, though significant heterogeneity emerged in certain subgroups. Gender-stratified analysis indicated that, with the exception of MUFA/FA, the effects and risk patterns of other FAs were generally consistent across both men and women, with no significant interaction observed (Supplementary Table S6). Notably, MUFA/FA presented a higher NSCLC risk in women (*P* for interaction = 0.044). Furthermore, significant heterogeneity was detected in BMI subgroups for LA, LA/FA, omega-6, and PUFA, suggesting stronger protective effects of these FAs against NSCLC in individuals with obesity (Supplementary Table S7). In contrast, no significant interactions were identified within subgroups based on age, family history of cancer, race, or history of DM (Supplementary Tables S8–S11). Interaction effects were identified exclusively for omega-3 (*p* = 0.03), LA/FA (*p* = 0.021), and DHA/FA (*p* = 0.006) within subgroups categorized by lipid-lowering medication use, cardiovascular disease history, and alcohol consumption (Supplementary Tables S12–S14). Notably, within the smoking-status subgroup, several FAs demonstrated significant heterogeneity. Specifically, LA/FA, omega-6/FA, PUFA/MUFA, and PUFA/FA were associated with an increased NSCLC risk in never-smokers but significantly reduced risk in former smokers. Conversely, MUFA/FA and SFA/FA were protective in never-smokers yet significantly elevated the NSCLC risk in current smokers (Supplementary Table S15).

## Discussion

This study provided an in-depth analysis of the influence of various FAs and their ratios on NSCLC risk. The results demonstrated a significant inverse relationship between DHA, LA, omega-3, and PUFA with NSCLC risk, while higher MUFA/FA and SFA/FA ratios correlated with increased risk. Stratified analysis highlighted the modifying effects of obesity, gender, and smoking status on these associations, with pronounced impacts observed among smokers and individuals with obesity. Additionally, DHA, LA, and omega-3/FA were associated with a delayed onset of NSCLC, whereas elevated MUFA/FA and SFA/FA ratios were linked to an earlier onset. This evidence not only advances the understanding of FA roles in NSCLC prevention but also suggests that modulating FA levels could be a strategic intervention in oncology for reducing and delaying NSCLC onset.

Extensive research has established the relationship between FAs and cancer, particularly the anti-inflammatory properties of omega-3 FAs and the pro-inflammatory nature of omega-6 FAs. Calder’s review highlighted omega-3’s role in reducing inflammation and its potential anticancer effects by inhibiting the release of pro-inflammatory cytokines and interleukins (19). Larsson et al. demonstrated that long-chain omega-3 FAs might modulate cancer cell proliferation and apoptosis by altering the FA composition of cell membranes (7). The results of this study align with previous research, affirming the protective roles of DHA, LA, and other omega-3 and omega-6 FAs in mitigating NSCLC risk. Additionally, the study identified a correlation between elevated MUFA/FA and SFA/FA levels and increased NSCLC risk, consistent with Serini et al.’s (20) findings, which suggest that saturated FAs may contribute to tumor growth (21).

This study enhances the comprehension of the intricate relationship between FAs and NSCLC. AFT model results indicated that elevated levels of DHA, LA, and PUFA not only diminished the overall NSCLC risk but also significantly delayed its onset, highlighting the protective role of FAs in NSCLC prevention and management. Moreover, RCS analysis identified a non-linear association between omega-3/FA and PUFA/FA ratios and NSCLC risk, suggesting that moderate omega-3 FA intake is essential for reducing NSCLC risk, with diminishing returns beyond a certain threshold. Consequently, dietary guidelines should prioritize adequate FA intake to maximize health benefits. This conclusion is consistent with previous studies that have highlighted the anti-inflammatory and anticancer properties of omega-3 FAs, emphasizing the importance of optimal intake levels [5, 7]. This study recommends a daily intake of at least 1.6 g of Omega-3 fatty acids for men and 1.1 g for women, primarily derived from Omega-3-rich fish or plant-based foods. PUFAs should comprise 5–10% of total energy intake, sourced from vegetable oils and nuts. Concurrently, SFA intake should be limited to less than 10% of total energy, with a focus on reducing the consumption of red meat and full-fat dairy products. A moderate intake of MUFAs, primarily from healthy vegetable oils and nuts, is also recommended. These dietary guidelines are designed to optimize fatty acid intake ratios and potentially reduce NSCLC risk.

Notable heterogeneity in the effects of FAs across subgroups was identified. Elevated PUFA levels correlated with a reduced NSCLC risk among smokers, a relationship that was not statistically significant in non-smokers, implying that smoking may modulate FA metabolism or alter their cancer-related pathways (22). Furthermore, variations in the protective effects and risk associations of FAs were observed across genders and BMI categories, aligning with Calder’s (5) findings on metabolic differences in FAs across diverse populations. These data emphasize the complex role of FAs in NSCLC risk, underscoring the necessity for personalized dietary recommendations.

Mechanistic studies on NSCLC indicate that the anti-inflammatory properties of DHA and LA may influence both the onset and progression of NSCLC through multiple pathways. Cheng et al. (23) demonstrated that DHA and omega-3 inhibited the synthesis of key inflammatory mediators, including leukotrienes and prostaglandins, which were integral to inflammation and cancer progression (24). Yin et al. (25) further validated that DHA might suppress inflammation-related signaling pathways, such as the nuclear factor κB (NF-κB) pathway, thereby reducing cell proliferation, promoting apoptosis, and inhibiting tumor growth (26).

Conversely, LA may contribute to NSCLC tumor cell proliferation by generating pro-inflammatory metabolites like arachidonic acid (27, 28). Additionally, FAs influence membrane fluidity and function by altering the FA composition of cell membranes, potentially playing a role in the initiation and progression of NSCLC (29). Research indicates that DHA and omega-3 can incorporate into cell membranes, modifying their fluidity and microdomain structure, which in turn affects membrane protein function and signal transduction pathways (30, 31). These alterations may impact cell proliferation, apoptosis, and metabolic pathways, thereby modulating cancer cell growth and migration to some extent.

The MUFA and SFA may modulate NSCLC risk through distinct metabolic pathways. Elevated SFA levels are implicated in the activation of pro-inflammatory mechanisms, such as Toll-like receptor 4, which intensify inflammatory responses (32). Excessive MUFA and SFA intake can result in lipid peroxidation, generating reactive oxygen species that elevate DNA damage and cancer risk (33). Additionally, FA metabolites may influence NSCLC onset and progression by modifying the tumor microenvironment. For example, metabolites of PUFA, including lipoxins and eicosanoids, exhibit anti-inflammatory properties that regulate immune cell activity, inhibit tumor angiogenesis, and suppress cell invasion within the tumor microenvironment (34–36).

The study’s strength lies in its large-scale prospective design, leveraging long-term follow-up data that enhances both statistical significance and result robustness. The use of precise NMR measurements mitigates recall bias typically associated with dietary questionnaires, thereby bolstering the reliability of the conclusions. The comprehensive analysis of diverse FA profiles, including the impact of specific FAs and their ratios on NSCLC risk, offers an in-depth understanding of the intricate relationship between FAs and NSCLC risk. Employing multiple statistical models and conducting sensitivity analyses further reinforce the robustness of the findings, ensuring a high degree of confidence in the conclusions. However, as an observational study, it remains subject to potential confounding factors. Moreover, reliance on a single FA measurement may not fully capture long-term FA exposure, potentially limiting the findings’ accuracy in reflecting prolonged dietary patterns. The lack of mechanistic studies in this research restricts a thorough understanding of the relationship between FAs and NSCLC risk. Future investigations should focus on elucidating the underlying biological mechanisms. Additionally, information bias and heterogeneity within subgroup analyses may impact result interpretation, and the external validity of the results remains limited. Consequently, while the study emphasizes the significant role of FA levels in NSCLC risk, causal relationships require further validation through randomized controlled trials. Replicating these analyses across diverse regions and populations is essential to confirm the generalizability and broader applicability of the results.

## Conclusion

This study provides a detailed analysis of the impact of distinct FAs on NSCLC risk, revealing that elevated concentrations of DHA, omega-3, and LA are markedly linked to a reduced risk of NSCLC, whereas higher MUFA/FA and SFA/FA levels correlate with an increased risk. The involvement of these FAs in NSCLC pathogenesis may occur through mechanisms involving inflammatory modulation, alterations in cell membrane dynamics and signaling pathways, and the regulation of the tumor microenvironment. This study offers novel perspectives on the role of FAs in NSCLC prevention and emphasizes the potential impact of dietary FA composition on NSCLC risk. The results indicate that modifying dietary FA composition may be an effective strategy for reducing NSCLC incidence.

## Data Availability

The raw data supporting the conclusions of this article will be made available by the authors, without undue reservation.

## Footnotes

^1^ https://biobank.ndph.ox.ac.uk/showcase/ukb/docs/NMR_companion_phase2.pdf

## Glossary

NSCLC: Non-small cell lung cancer
Fas: Fatty acids
AFT: Accelerated failure time
RCS: Restricted cubic spline
DHA: Docosahexaenoic acid
LA: Linoleic acid
UKB: UK Biobank
NMR: Nuclear magnetic resonance
MUFA: Monounsaturated fatty acids
Omega-3: Omega-3 FAs
Omega-6 FAs: Omega-6
PUFA: Polyunsaturated fatty acids
SFA: Saturated FAs
ICD-10: International Classification of Diseases, 10th Revision
BMI: Body mass index
TDI: Townsend deprivation index
CVD: Cardiovascular disease
MET: Metabolic equivalent task
DM: Diabetes mellitus
HRs: Hazard ratios
CIs: Confidence intervals
SD: Standard deviation
NF-κB: Nuclear factor κB
