# Supplementary material for: Plasma fatty acid levels and risk of non-small cell lung cancer: a large-scale prospective cohort study
Source: Front Nutr. 2024 Sep 18;11:1462300. doi: 10.3389/fnut.2024.1462300 (PMC11457710; doi:10.3389/fnut.2024.1462300)

**Plasma Fatty Acid Levels and Risk of Non-Small Cell Lung Cancer: A Large-Scale Prospective Cohort Study**

**HuaLong Lin^1^, Qiu-Yan Lin^1^, Jie-Ni Feng^1^, Wei-E Zheng^2^, Chuang Yang^3^ and Shao-Fei Yuan^1^***

[Table S1. Definition of NSCLC in UK Biobank. 2](#_Toc171333469)

[Table S2. Adjusted median time difference for NSCLC occurrence in the second, third and fourth quintile groups compared to the 14 FAs lowest quintile (Q1) groups. 3](#_Toc171333470)

[Table S3. The association between circulating fatty acids and the risk of NSCLC after excluding participants with follow-up within 2 years. 4](#_Toc171333471)

[Table S4. The association between circulating fatty acids and the risk of NSCLC after excluding participants with any covariates missing values at baseline. 5](#_Toc171333472)

[Table S5. The association between circulating fatty acids and the risk of NSCLC after multiple imputations of other four data sets. 6](#_Toc171333473)

[Table S6. The association between circulating fatty acids and the risk of NSCLC across sex. 7](#_Toc171333474)

[Table S7. The association between circulating fatty acids and the risk of NSCLC across BMI. 8](#_Toc171333475)

[Table S8. The association between circulating fatty acids and the risk of NSCLC across age. 9](#_Toc171333476)

[Table S9. The association between circulating fatty acids and the risk of NSCLC across the history of cancer family. 10](#_Toc171333477)

[Table S10. The association between circulating fatty acids and the risk of NSCLC across the history of race. 11](#_Toc171333478)

[Table S11. The association between circulating fatty acids and the risk of NSCLC across the history of DM. 12](#_Toc171333479)

[Table S12. The association between circulating fatty acids and the risk of NSCLC across the history of lowing lipid drugs. 13](#_Toc171333480)

[Table S13. The association between circulating fatty acids and the risk of NSCLC across the history of CVD. 14](#_Toc171333481)

[Table S14. The association between circulating fatty acids and the risk of NSCLC across drinking status. 15](#_Toc171333482)

[Table S15. The association between circulating fatty acids and the risk of NSCLC across smoking status. 16](#_Toc171333483)

[Appendix 1. Proof of Language Editing 17](#_Toc171333484)

| Table S1. Definition of NSCLC in UK Biobank. | | |
| --- | --- | --- |
| **Cancer Type** | **ICD-10** | **UK Biobank data field 40011 values** |
| NSCLC | C34 | 8012, 8013, 8020, 8022, 8032, 8033, 8046, 8050, 8070, 8071, 8072, 8074, 8140, 8230, 8244, 8250, 8253, 8260, 8310, 8440, 8480, 8481, 8490, 8550, 8560, 8574, 8800, 8972, 8980, 9040, 9120,8255 |

NSCLC: non-small cell lung cancer

| Table S2. Adjusted median time difference for NSCLC occurrence in the second, third and fourth quintile groups compared to the 14 FAs lowest quintile (Q1) groups. | | | | | | | | | | | |
| --- | --- | --- | --- | --- | --- | --- | --- | --- | --- | --- | --- |
| **Type** | **Q1** | **Q2** | | | **Q3** | | | **Q4** | | |  |
|  |  | **median** | **95% CI low** | **95% CI high** | **median** | **95% CI low** | **95% CI high** | **median** | **95% CI low** | **95% CI high** | ***P* for trend** |
| DHA | Reference | -19.79 | 10.67 | -48.76 | -35.69 | -4.18 | -65.56 | -65.06 | -32.31 | -95.95 | <0.001 |
| DHA/FA | Reference | -31.2 | -3.95 | -57.13 | -54.82 | -26.33 | -81.81 | -65.48 | -35.06 | -94.17 | <0.001 |
| LA | Reference | -35.86 | -5.24 | -64.94 | -47.07 | -15.41 | -77.05 | -79.62 | -47.6 | -109.83 | <0.001 |
| LA/FA | Reference | -42.31 | -19.75 | -63.79 | -49.39 | -24.88 | -72.62 | -88.86 | -62.12 | -113.94 | <0.001 |
| MUFA/FA | Reference | 63.28 | 108.03 | 21.26 | 56.55 | 100.91 | 14.9 | 108.15 | 155.77 | 63.45 | <0.001 |
| Omega-3 | Reference | -19.2 | 12.96 | -49.77 | -52.39 | -20.06 | -83.01 | -47.5 | -14.08 | -79.11 | <0.001 |
| Omega-3/FA | Reference | -17.93 | 12.78 | -47.12 | -31.02 | 0.37 | -60.79 | -69.7 | -37.19 | -100.36 | <0.001 |
| Omega-6 | Reference | -27.76 | 3.8 | -57.74 | -56.17 | -23.85 | -86.76 | -63.93 | -30.93 | -95.1 | <0.001 |
| Omega-6/Omega-3 | Reference | 6.4 | 44.35 | -29.56 | 19.45 | 58.66 | -17.68 | 41.95 | 83.22 | 2.92 | 0.025 |
| Omega-6/FA | Reference | -10.55 | 16.19 | -36.04 | -34.96 | -6.72 | -61.75 | -55.38 | -24.11 | -84.8 | 0.005 |
| PUFA | Reference | -2.79 | 30.25 | -34.19 | -38.3 | -4.79 | -70.04 | -68.79 | -34.87 | -100.8 | <0.001 |
| PUFA/MUFA | Reference | -34.14 | -9.87 | -57.26 | -35.25 | -9.09 | -60.07 | -77.78 | -48.54 | -105.21 | <0.001 |
| PUFA/FA | Reference | -16.74 | 7.54 | -39.89 | -35.25 | -9.15 | -60.01 | -77.98 | -48.9 | -105.25 | <0.001 |
| SFA/FA | Reference | 54.62 | 96.97 | 14.72 | 36.17 | 77.34 | -2.62 | 86.66 | 128.45 | 47.15 | <0.001 |

Models were adjusted with age, sex, race, BMI (body mass index), MET (metabolic equivalent task), TDI (Townsend Deprivation Index), smoking and drinking status, diet score, fasting time, family history of cancer, lipid-lowering drugs, insulin, and history of chronic diseases including DM (diabetes mellitus) and CVD (cardiovascular disease). NSCLC: non-small cell lung cancer; DHA: docosahexaenoic acid; DHA/FA: docosahexaenoic acid to total fatty acids percentage; LA: linoleic acid; LA/FA: linoleic acid to total fatty acids percentage; MUFA: monounsaturated fatty acids; MUFA/FA: monounsaturated fatty acids to total fatty acids percentage; Omega-3: Omega-3 fatty acids; Omega-3/FA: Omega-3 fatty acids to total fatty acids percentage; Omega-6: Omega-6 fatty acids; Omega-6/Omega-3: Omega-6 fatty acids to Omega-3 fatty acids ratio; Omega-6/FA: Omega-6 fatty acids to total fatty acids percentage; PUFA: polyunsaturated fatty acids; PUFA/MUFA: Polyunsaturated fatty acids to monounsaturated fatty acids ratio; PUFA/FA: polyunsaturated fatty acids to total fatty acids percentage; SFA: saturated fatty acids; SFA/FA: saturated fatty acids to total fatty acids percentage; FA: total fatty acids;

| Table S3. The association between circulating fatty acids and the risk of NSCLC after excluding participants with follow-up within 2 years. | | |
| --- | --- | --- |
| **Type** | **HR (95% CI)** | ***P*** |
| DHA | 0.9 (0.85-0.96) | 0.002 |
| DHA/FA | 0.9 (0.85-0.96) | 0.001 |
| LA | 0.9 (0.84-0.95) | <0.001 |
| LA/FA | 0.84 (0.79-0.89) | <0.001 |
| MUFA/FA | 1.13 (1.06-1.2) | <0.001 |
| Omega-3 | 0.92 (0.86-0.97) | 0.005 |
| Omega-3/FA | 0.9 (0.84-0.96) | <0.001 |
| Omega-6 | 0.91 (0.86-0.97) | 0.003 |
| Omega-6/Omega-3 | 1.08 (1.04-1.12) | <0.001 |
| Omega-6/FA | 0.89 (0.84-0.94) | <0.001 |
| PUFA | 0.9 (0.85-0.96) | 0.001 |
| PUFA/MUFA | 0.85 (0.8-0.91) | <0.001 |
| PUFA/FA | 0.86 (0.81-0.91) | <0.001 |
| SFA/FA | 1.13 (1.07-1.19) | <0.001 |

Models were adjusted with age, sex, race, BMI (body mass index), MET (metabolic equivalent task), TDI (Townsend Deprivation Index), smoking and drinking status, diet score, fasting time, family history of cancer, lipid-lowering drugs, insulin, and history of chronic diseases including DM (diabetes mellitus) and CVD (cardiovascular disease). NSCLC: non-small cell lung cancer; DHA: docosahexaenoic acid; DHA/FA: docosahexaenoic acid to total fatty acids percentage; LA: linoleic acid; LA/FA: linoleic acid to total fatty acids percentage; MUFA: monounsaturated fatty acids; MUFA/FA: monounsaturated fatty acids to total fatty acids percentage; Omega-3: Omega-3 fatty acids; Omega-3/FA: Omega-3 fatty acids to total fatty acids percentage; Omega-6: Omega-6 fatty acids; Omega-6/Omega-3: Omega-6 fatty acids to Omega-3 fatty acids ratio; Omega-6/FA: Omega-6 fatty acids to total fatty acids percentage; PUFA: polyunsaturated fatty acids; PUFA/MUFA: Polyunsaturated fatty acids to monounsaturated fatty acids ratio; PUFA/FA: polyunsaturated fatty acids to total fatty acids percentage; SFA: saturated fatty acids; SFA/FA: saturated fatty acids to total fatty acids percentage; FA: total fatty acids;

| Table S4. The association between circulating fatty acids and the risk of NSCLC after excluding participants with any covariates missing values at baseline. | | |
| --- | --- | --- |
| **Type** | **HR (95% CI)** | ***P*** |
| DHA | 0.89 (0.83-0.96) | 0.001 |
| DHA/FA | 0.88 (0.82-0.95) | <0.001 |
| LA | 0.88 (0.82-0.94) | <0.001 |
| LA/FA | 0.83 (0.77-0.88) | <0.001 |
| MUFA/FA | 1.15 (1.07-1.22) | <0.001 |
| Omega-3 | 0.91 (0.85-0.97) | 0.005 |
| Omega-3/FA | 0.89 (0.83-0.95) | 0.001 |
| Omega-6 | 0.9 (0.84-0.97) | 0.003 |
| Omega-6/Omega-3 | 1.08 (1.04-1.12) | <0.001 |
| Omega-6/FA | 0.88 (0.83-0.94) | <0.001 |
| PUFA | 0.89 (0.83-0.96) | 0.001 |
| PUFA/MUFA | 0.83 (0.78-0.9) | <0.001 |
| PUFA/FA | 0.85 (0.8-0.9) | <0.001 |
| SFA/FA | 1.13 (1.06-1.2) | <0.001 |

Models were adjusted with age, sex, race, BMI (body mass index), MET (metabolic equivalent task), TDI (Townsend Deprivation Index), smoking and drinking status, diet score, fasting time, family history of cancer, lipid-lowering drugs, insulin, and history of chronic diseases including DM (diabetes mellitus) and CVD (cardiovascular disease). NSCLC: non-small cell lung cancer; DHA: docosahexaenoic acid; DHA/FA: docosahexaenoic acid to total fatty acids percentage; LA: linoleic acid; LA/FA: linoleic acid to total fatty acids percentage; MUFA: monounsaturated fatty acids; MUFA/FA: monounsaturated fatty acids to total fatty acids percentage; Omega-3: Omega-3 fatty acids; Omega-3/FA: Omega-3 fatty acids to total fatty acids percentage; Omega-6: Omega-6 fatty acids; Omega-6/Omega-3: Omega-6 fatty acids to Omega-3 fatty acids ratio; Omega-6/FA: Omega-6 fatty acids to total fatty acids percentage; PUFA: polyunsaturated fatty acids; PUFA/MUFA: Polyunsaturated fatty acids to monounsaturated fatty acids ratio; PUFA/FA: polyunsaturated fatty acids to total fatty acids percentage; SFA: saturated fatty acids; SFA/FA: saturated fatty acids to total fatty acids percentage; FA: total fatty acids;

| Table S5. The association between circulating fatty acids and the risk of NSCLC after multiple imputations of other four data sets. | | | | | | |
| --- | --- | --- | --- | --- | --- | --- |
| **Type** | **Data set 1** | **Data set 2** | **Data set 3** | **Data set 4** | **Pooled results** | ***P*** |
|  | **HR (95% CI)** | **HR (95% CI)** | **HR (95% CI)** | **HR (95% CI)** | **HR (95% CI)** |  |
| DHA | 0.89 (0.84-0.95) | 0.89 (0.84-0.95) | 0.89 (0.84-0.95) | 0.89 (0.84-0.95) | 0.89 (0.84-0.95) | <0.001 |
| DHA/FA | 0.9 (0.85-0.96) | 0.9 (0.85-0.96) | 0.9 (0.85-0.96) | 0.9 (0.85-0.96) | 0.9 (0.85-0.96) | <0.001 |
| LA | 0.88 (0.83-0.93) | 0.87 (0.83-0.93) | 0.87 (0.83-0.93) | 0.88 (0.83-0.93) | 0.87 (0.83-0.93) | <0.001 |
| LA/FA | 0.84 (0.8-0.89) | 0.84 (0.79-0.89) | 0.84 (0.79-0.89) | 0.84 (0.8-0.89) | 0.84 (0.79-0.89) | <0.001 |
| MUFA/FA | 1.12 (1.06-1.18) | 1.12 (1.06-1.18) | 1.12 (1.06-1.18) | 1.12 (1.06-1.18) | 1.12 (1.06-1.18) | <0.001 |
| Omega-3 | 0.9 (0.85-0.96) | 0.9 (0.85-0.96) | 0.9 (0.85-0.96) | 0.9 (0.85-0.96) | 0.9 (0.85-0.96) | <0.001 |
| Omega-3/FA | 0.89 (0.84-0.95) | 0.89 (0.84-0.95) | 0.9 (0.84-0.95) | 0.89 (0.84-0.95) | 0.89 (0.84-0.95) | <0.001 |
| Omega-6 | 0.9 (0.85-0.95) | 0.89 (0.85-0.95) | 0.89 (0.85-0.95) | 0.9 (0.85-0.95) | 0.89 (0.85-0.95) | <0.001 |
| Omega-6/Omega-3 | 1.08 (1.04-1.11) | 1.08 (1.04-1.11) | 1.07 (1.04-1.11) | 1.08 (1.04-1.11) | 1.08 (1.04-1.11) | <0.001 |
| Omega-6/FA | 0.9 (0.86-0.95) | 0.9 (0.86-0.95) | 0.9 (0.86-0.95) | 0.9 (0.86-0.95) | 0.9 (0.86-0.95) | <0.001 |
| PUFA | 0.89 (0.84-0.94) | 0.89 (0.84-0.94) | 0.89 (0.84-0.94) | 0.89 (0.84-0.94) | 0.89 (0.84-0.94) | <0.001 |
| PUFA/MUFA | 0.86 (0.81-0.91) | 0.86 (0.81-0.91) | 0.86 (0.81-0.91) | 0.86 (0.81-0.91) | 0.86 (0.81-0.91) | <0.001 |
| PUFA/FA | 0.87 (0.83-0.92) | 0.87 (0.82-0.92) | 0.87 (0.82-0.92) | 0.87 (0.82-0.92) | 0.87 (0.82-0.92) | <0.001 |
| SFA/FA | 1.12 (1.06-1.17) | 1.12 (1.06-1.18) | 1.12 (1.06-1.17) | 1.12 (1.06-1.17) | 1.12 (1.06-1.18) | <0.001 |

Models were adjusted with age, sex, race, BMI (body mass index), MET (metabolic equivalent task), TDI (Townsend Deprivation Index), smoking and drinking status, diet score, fasting time, family history of cancer, lipid-lowering drugs, insulin, and history of chronic diseases including DM (diabetes mellitus) and CVD (cardiovascular disease). NSCLC: non-small cell lung cancer; DHA: docosahexaenoic acid; DHA/FA: docosahexaenoic acid to total fatty acids percentage; LA: linoleic acid; LA/FA: linoleic acid to total fatty acids percentage; MUFA: monounsaturated fatty acids; MUFA/FA: monounsaturated fatty acids to total fatty acids percentage; Omega-3: Omega-3 fatty acids; Omega-3/FA: Omega-3 fatty acids to total fatty acids percentage; Omega-6: Omega-6 fatty acids; Omega-6/Omega-3: Omega-6 fatty acids to Omega-3 fatty acids ratio; Omega-6/FA: Omega-6 fatty acids to total fatty acids percentage; PUFA: polyunsaturated fatty acids; PUFA/MUFA: Polyunsaturated fatty acids to monounsaturated fatty acids ratio; PUFA/FA: polyunsaturated fatty acids to total fatty acids percentage; SFA: saturated fatty acids; SFA/FA: saturated fatty acids to total fatty acids percentage; FA: total fatty acids

| Table S6. The association between circulating fatty acids and the risk of NSCLC across sex. | | | | | |
| --- | --- | --- | --- | --- | --- |
| **Type** | **Male** | | **Female** | | ***P* for interaction** |
|  | **HR (95% CI)** | **P** | **HR (95% CI)** | **P** |  |
| DHA | 0.89 (0.81-0.97) | 0.006 | 0.9 (0.83-0.98) | 0.012 | 0.723 |
| DHA/FA | 0.92 (0.85-1) | 0.059 | 0.88 (0.8-0.96) | 0.003 | 0.734 |
| LA | 0.84 (0.77-0.91) | <0.001 | 0.92 (0.85-1) | 0.044 | 0.3 |
| LA/FA | 0.86 (0.8-0.93) | <0.001 | 0.81 (0.74-0.88) | <0.001 | 0.402 |
| MUFA/FA | 1.05 (0.98-1.13) | 0.181 | 1.22 (1.12-1.32) | <0.001 | 0.044 |
| Omega-3 | 0.87 (0.8-0.94) | 0.001 | 0.94 (0.87-1.02) | 0.145 | 0.153 |
| Omega-3/FA | 0.88 (0.8-0.95) | 0.003 | 0.92 (0.84-1) | 0.04 | 0.276 |
| Omega-6 | 0.86 (0.79-0.93) | <0.001 | 0.94 (0.87-1.01) | 0.11 | 0.375 |
| Omega-6/Omega-3 | 1.08 (1.02-1.13) | 0.006 | 1.07 (1.02-1.13) | 0.009 | 0.886 |
| Omega-6/FA | 0.95 (0.88-1.01) | 0.119 | 0.84 (0.77-0.91) | <0.001 | 0.106 |
| PUFA | 0.85 (0.78-0.92) | <0.001 | 0.93 (0.86-1.01) | 0.078 | 0.268 |
| PUFA/MUFA | 0.91 (0.84-0.99) | 0.025 | 0.79 (0.73-0.87) | <0.001 | 0.115 |
| PUFA/FA | 0.91 (0.85-0.98) | 0.008 | 0.81 (0.75-0.88) | <0.001 | 0.19 |
| SFA/FA | 1.11 (1.04-1.19) | 0.002 | 1.12 (1.04-1.21) | 0.005 | 0.698 |

Models were adjusted with age, sex, race, BMI (body mass index), MET (metabolic equivalent task), TDI (Townsend Deprivation Index), smoking and drinking status, diet score, fasting time, family history of cancer, lipid-lowering drugs, insulin, and history of chronic diseases including DM (diabetes mellitus) and CVD (cardiovascular disease). NSCLC: non-small cell lung cancer; DHA: docosahexaenoic acid; DHA/FA: docosahexaenoic acid to total fatty acids percentage; LA: linoleic acid; LA/FA: linoleic acid to total fatty acids percentage; MUFA: monounsaturated fatty acids; MUFA/FA: monounsaturated fatty acids to total fatty acids percentage; Omega-3: Omega-3 fatty acids; Omega-3/FA: Omega-3 fatty acids to total fatty acids percentage; Omega-6: Omega-6 fatty acids; Omega-6/Omega-3: Omega-6 fatty acids to Omega-3 fatty acids ratio; Omega-6/FA: Omega-6 fatty acids to total fatty acids percentage; PUFA: polyunsaturated fatty acids; PUFA/MUFA: Polyunsaturated fatty acids to monounsaturated fatty acids ratio; PUFA/FA: polyunsaturated fatty acids to total fatty acids percentage; SFA: saturated fatty acids; SFA/FA: saturated fatty acids to total fatty acids percentage; FA: total fatty acids;

| Table S7. The association between circulating fatty acids and the risk of NSCLC across BMI. | | | | | |
| --- | --- | --- | --- | --- | --- |
| **Type** | **< 30 kg/m2** | | **≥ 30 kg/m2** | | ***P* for interaction** |
|  | **HR (95% CI)** | ***P*** | **HR (95% CI)** | ***P*** |  |
| DHA | 0.89 (0.83-0.96) | 0.001 | 0.91 (0.81-1.03) | 0.147 | 0.356 |
| DHA/FA | 0.91 (0.85-0.97) | 0.005 | 0.9 (0.8-1.02) | 0.11 | 0.801 |
| LA | 0.85 (0.79-0.91) | <0.001 | 0.98 (0.87-1.1) | 0.68 | 0.007 |
| LA/FA | 0.8 (0.75-0.86) | <0.001 | 0.95 (0.85-1.07) | 0.413 | 0.004 |
| MUFA/FA | 1.15 (1.08-1.22) | <0.001 | 1.05 (0.94-1.17) | 0.366 | 0.07 |
| Omega-3 | 0.92 (0.86-0.98) | 0.014 | 0.89 (0.79-1) | 0.045 | 0.975 |
| Omega-3/FA | 0.92 (0.86-0.98) | 0.013 | 0.84 (0.74-0.96) | 0.012 | 0.467 |
| Omega-6 | 0.87 (0.81-0.93) | <0.001 | 0.99 (0.88-1.11) | 0.834 | 0.013 |
| Omega-6/Omega-3 | 1.08 (1.02-1.13) | 0.004 | 1.06 (1-1.13) | 0.067 | 0.582 |
| Omega-6/FA | 0.87 (0.82-0.93) | <0.001 | 0.97 (0.88-1.08) | 0.583 | 0.07 |
| PUFA | 0.87 (0.81-0.93) | <0.001 | 0.96 (0.86-1.07) | 0.469 | 0.028 |
| PUFA/MUFA | 0.84 (0.78-0.9) | <0.001 | 0.92 (0.82-1.04) | 0.202 | 0.124 |
| PUFA/FA | 0.85 (0.8-0.9) | <0.001 | 0.93 (0.84-1.03) | 0.155 | 0.097 |
| SFA/FA | 1.13 (1.06-1.19) | <0.001 | 1.08 (0.98-1.19) | 0.135 | 0.636 |

Models were adjusted with age, sex, race, BMI (body mass index), MET (metabolic equivalent task), TDI (Townsend Deprivation Index), smoking and drinking status, diet score, fasting time, family history of cancer, lipid-lowering drugs, insulin, and history of chronic diseases including DM (diabetes mellitus) and CVD (cardiovascular disease). NSCLC: non-small cell lung cancer; DHA: docosahexaenoic acid; DHA/FA: docosahexaenoic acid to total fatty acids percentage; LA: linoleic acid; LA/FA: linoleic acid to total fatty acids percentage; MUFA: monounsaturated fatty acids; MUFA/FA: monounsaturated fatty acids to total fatty acids percentage; Omega-3: Omega-3 fatty acids; Omega-3/FA: Omega-3 fatty acids to total fatty acids percentage; Omega-6: Omega-6 fatty acids; Omega-6/Omega-3: Omega-6 fatty acids to Omega-3 fatty acids ratio; Omega-6/FA: Omega-6 fatty acids to total fatty acids percentage; PUFA: polyunsaturated fatty acids; PUFA/MUFA: Polyunsaturated fatty acids to monounsaturated fatty acids ratio; PUFA/FA: polyunsaturated fatty acids to total fatty acids percentage; SFA: saturated fatty acids; SFA/FA: saturated fatty acids to total fatty acids percentage; FA: total fatty acids;

| Table S8. The association between circulating fatty acids and the risk of NSCLC across age. | | | | | |
| --- | --- | --- | --- | --- | --- |
| **Type** | **< 60 years** | | **≥60 years** | | ***P* for interaction** |
|  | **HR (95% CI)** | ***P*** | **HR (95% CI)** | ***P*** |  |
| DHA | 0.9 (0.8-1.01) | 0.066 | 0.89 (0.83-0.95) | <0.001 | 0.718 |
| DHA/FA | 0.9 (0.81-1.01) | 0.077 | 0.9 (0.84-0.97) | 0.004 | 0.951 |
| LA | 0.87 (0.79-0.96) | 0.007 | 0.87 (0.81-0.94) | <0.001 | 0.563 |
| LA/FA | 0.83 (0.75-0.91) | <0.001 | 0.84 (0.79-0.9) | <0.001 | 0.963 |
| MUFA/FA | 1.18 (1.07-1.3) | 0.001 | 1.1 (1.03-1.17) | 0.006 | 0.371 |
| Omega-3 | 0.94 (0.85-1.04) | 0.253 | 0.89 (0.83-0.95) | <0.001 | 0.274 |
| Omega-3/FA | 0.92 (0.83-1.03) | 0.156 | 0.89 (0.83-0.95) | <0.001 | 0.546 |
| Omega-6 | 0.89 (0.8-0.98) | 0.015 | 0.89 (0.83-0.96) | 0.002 | 0.627 |
| Omega-6/Omega-3 | 1.09 (1.04-1.14) | <0.001 | 1.06 (1.01-1.12) | 0.018 | 0.477 |
| Omega-6/FA | 0.88 (0.8-0.96) | 0.006 | 0.91 (0.85-0.97) | 0.004 | 0.595 |
| PUFA | 0.89 (0.8-0.98) | 0.018 | 0.88 (0.82-0.94) | <0.001 | 0.456 |
| PUFA/MUFA | 0.83 (0.74-0.92) | <0.001 | 0.87 (0.81-0.93) | <0.001 | 0.533 |
| PUFA/FA | 0.85 (0.78-0.94) | 0.001 | 0.87 (0.82-0.93) | <0.001 | 0.81 |
| SFA/FA | 1.08 (0.99-1.18) | 0.088 | 1.14 (1.07-1.21) | <0.001 | 0.407 |

Models were adjusted with age, sex, race, BMI (body mass index), MET (metabolic equivalent task), TDI (Townsend Deprivation Index), smoking and drinking status, diet score, fasting time, family history of cancer, lipid-lowering drugs, insulin, and history of chronic diseases including DM (diabetes mellitus) and CVD (cardiovascular disease). NSCLC: non-small cell lung cancer; DHA: docosahexaenoic acid; DHA/FA: docosahexaenoic acid to total fatty acids percentage; LA: linoleic acid; LA/FA: linoleic acid to total fatty acids percentage; MUFA: monounsaturated fatty acids; MUFA/FA: monounsaturated fatty acids to total fatty acids percentage; Omega-3: Omega-3 fatty acids; Omega-3/FA: Omega-3 fatty acids to total fatty acids percentage; Omega-6: Omega-6 fatty acids; Omega-6/Omega-3: Omega-6 fatty acids to Omega-3 fatty acids ratio; Omega-6/FA: Omega-6 fatty acids to total fatty acids percentage; PUFA: polyunsaturated fatty acids; PUFA/MUFA: Polyunsaturated fatty acids to monounsaturated fatty acids ratio; PUFA/FA: polyunsaturated fatty acids to total fatty acids percentage; SFA: saturated fatty acids; SFA/FA: saturated fatty acids to total fatty acids percentage; FA: total fatty acids;

| Table S9. The association between circulating fatty acids and the risk of NSCLC across the history of cancer family. | | | | | |
| --- | --- | --- | --- | --- | --- |
| **Type** | **Yes** |  | **No** |  | ***P* for interaction** |
|  | **HR (95% CI)** | ***P*** | **HR (95% CI)** | ***P*** |  |
| DHA | 0.95 (0.87-1.05) | 0.345 | 0.86 (0.8-0.93) | <0.001 | 0.311 |
| DHA/FA | 0.96 (0.87-1.06) | 0.47 | 0.87 (0.81-0.94) | <0.001 | 0.516 |
| LA | 0.83 (0.75-0.92) | <0.001 | 0.9 (0.84-0.97) | 0.004 | 0.874 |
| LA/FA | 0.8 (0.73-0.88) | <0.001 | 0.86 (0.8-0.92) | <0.001 | 0.786 |
| MUFA/FA | 1.08 (0.98-1.19) | 0.104 | 1.14 (1.07-1.22) | <0.001 | 0.43 |
| Omega-3 | 0.95 (0.87-1.05) | 0.308 | 0.88 (0.82-0.95) | <0.001 | 0.505 |
| Omega-3/FA | 0.96 (0.87-1.06) | 0.401 | 0.86 (0.8-0.93) | <0.001 | 0.576 |
| Omega-6 | 0.87 (0.79-0.96) | 0.006 | 0.91 (0.85-0.97) | 0.007 | 0.782 |
| Omega-6/Omega-3 | 1.07 (1-1.15) | 0.037 | 1.07 (1.03-1.12) | 0.001 | 0.531 |
| Omega-6/FA | 0.9 (0.82-0.98) | 0.016 | 0.9 (0.84-0.96) | 0.002 | 0.913 |
| PUFA | 0.88 (0.8-0.97) | 0.009 | 0.89 (0.83-0.96) | 0.001 | 0.679 |
| PUFA/MUFA | 0.88 (0.79-0.97) | 0.01 | 0.85 (0.79-0.91) | <0.001 | 0.707 |
| PUFA/FA | 0.88 (0.81-0.97) | 0.007 | 0.86 (0.8-0.92) | <0.001 | 0.768 |
| SFA/FA | 1.13 (1.04-1.23) | 0.005 | 1.11 (1.05-1.18) | <0.001 | 0.611 |

Models were adjusted with age, sex, race, BMI (body mass index), MET (metabolic equivalent task), TDI (Townsend Deprivation Index), smoking and drinking status, diet score, fasting time, family history of cancer, lipid-lowering drugs, insulin, and history of chronic diseases including DM (diabetes mellitus) and CVD (cardiovascular disease). NSCLC: non-small cell lung cancer; DHA: docosahexaenoic acid; DHA/FA: docosahexaenoic acid to total fatty acids percentage; LA: linoleic acid; LA/FA: linoleic acid to total fatty acids percentage; MUFA: monounsaturated fatty acids; MUFA/FA: monounsaturated fatty acids to total fatty acids percentage; Omega-3: Omega-3 fatty acids; Omega-3/FA: Omega-3 fatty acids to total fatty acids percentage; Omega-6: Omega-6 fatty acids; Omega-6/Omega-3: Omega-6 fatty acids to Omega-3 fatty acids ratio; Omega-6/FA: Omega-6 fatty acids to total fatty acids percentage; PUFA: polyunsaturated fatty acids; PUFA/MUFA: Polyunsaturated fatty acids to monounsaturated fatty acids ratio; PUFA/FA: polyunsaturated fatty acids to total fatty acids percentage; SFA: saturated fatty acids; SFA/FA: saturated fatty acids to total fatty acids percentage; FA: total fatty acids;

| Table S10. The association between circulating fatty acids and the risk of NSCLC across the history of race. | | | | | |
| --- | --- | --- | --- | --- | --- |
| **Type** | **White** |  | **Others** |  | ***P* for interaction** |
|  | **HR (95% CI)** | ***P*** | **HR (95% CI)** | ***P*** |  |
| DHA | 0.89 (0.84-0.95) | <0.001 | 0.89 (0.67-1.19) | 0.44 | 0.858 |
| DHA/FA | 0.9 (0.85-0.96) | <0.001 | 0.92 (0.71-1.2) | 0.542 | 0.705 |
| LA | 0.88 (0.82-0.93) | <0.001 | 0.86 (0.66-1.12) | 0.255 | 0.763 |
| LA/FA | 0.84 (0.79-0.89) | <0.001 | 0.86 (0.66-1.12) | 0.255 | 0.301 |
| MUFA/FA | 1.12 (1.06-1.18) | <0.001 | 1.15 (0.87-1.53) | 0.321 | 0.696 |
| Omega-3 | 0.91 (0.85-0.96) | 0.001 | 0.9 (0.68-1.19) | 0.462 | 0.986 |
| Omega-3/FA | 0.9 (0.84-0.95) | <0.001 | 0.91 (0.7-1.18) | 0.478 | 0.859 |
| Omega-6 | 0.9 (0.85-0.95) | <0.001 | 0.84 (0.64-1.1) | 0.208 | 0.926 |
| Omega-6/Omega-3 | 1.06 (1.01-1.11) | 0.008 | 1.12 (1.07-1.18) | <0.001 | 0.143 |
| Omega-6/FA | 0.9 (0.86-0.95) | <0.001 | 0.85 (0.66-1.11) | 0.228 | 0.766 |
| PUFA | 0.89 (0.84-0.94) | <0.001 | 0.83 (0.63-1.1) | 0.194 | 0.905 |
| PUFA/MUFA | 0.86 (0.81-0.91) | <0.001 | 0.82 (0.62-1.08) | 0.162 | 0.711 |
| PUFA/FA | 0.87 (0.82-0.92) | <0.001 | 0.82 (0.63-1.06) | 0.131 | 0.773 |
| SFA/FA | 1.11 (1.06-1.17) | <0.001 | 1.2 (0.94-1.52) | 0.143 | 0.964 |

Models were adjusted with age, sex, race, BMI (body mass index), MET (metabolic equivalent task), TDI (Townsend Deprivation Index), smoking and drinking status, diet score, fasting time, family history of cancer, lipid-lowering drugs, insulin, and history of chronic diseases including DM (diabetes mellitus) and CVD (cardiovascular disease). NSCLC: non-small cell lung cancer; DHA: docosahexaenoic acid; DHA/FA: docosahexaenoic acid to total fatty acids percentage; LA: linoleic acid; LA/FA: linoleic acid to total fatty acids percentage; MUFA: monounsaturated fatty acids; MUFA/FA: monounsaturated fatty acids to total fatty acids percentage; Omega-3: Omega-3 fatty acids; Omega-3/FA: Omega-3 fatty acids to total fatty acids percentage; Omega-6: Omega-6 fatty acids; Omega-6/Omega-3: Omega-6 fatty acids to Omega-3 fatty acids ratio; Omega-6/FA: Omega-6 fatty acids to total fatty acids percentage; PUFA: polyunsaturated fatty acids; PUFA/MUFA: Polyunsaturated fatty acids to monounsaturated fatty acids ratio; PUFA/FA: polyunsaturated fatty acids to total fatty acids percentage; SFA: saturated fatty acids; SFA/FA: saturated fatty acids to total fatty acids percentage; FA: total fatty acids;

| Table S11. The association between circulating fatty acids and the risk of NSCLC across the history of DM. | | | | | |
| --- | --- | --- | --- | --- | --- |
| **Type** | **Yes** |  | **No** |  | ***P* for interaction** |
|  | **HR (95% CI)** | ***P*** | **HR (95% CI)** | ***P*** |  |
| DHA | 0.95 (0.78-1.17) | 0.635 | 0.89 (0.83-0.94) | <0.001 | 0.329 |
| DHA/FA | 0.91 (0.75-1.11) | 0.347 | 0.9 (0.85-0.96) | 0.001 | 0.504 |
| LA | 0.91 (0.74-1.11) | 0.348 | 0.87 (0.82-0.93) | <0.001 | 0.574 |
| LA/FA | 0.8 (0.67-0.95) | 0.01 | 0.84 (0.8-0.9) | <0.001 | 0.805 |
| MUFA/FA | 1.12 (0.95-1.32) | 0.168 | 1.12 (1.06-1.19) | <0.001 | 0.347 |
| Omega-3 | 0.98 (0.81-1.18) | 0.796 | 0.9 (0.85-0.96) | <0.001 | 0.424 |
| Omega-3/FA | 0.95 (0.79-1.15) | 0.62 | 0.89 (0.84-0.95) | <0.001 | 0.416 |
| Omega-6 | 0.97 (0.8-1.17) | 0.734 | 0.89 (0.84-0.94) | <0.001 | 0.374 |
| Omega-6/Omega-3 | 1.08 (0.92-1.27) | 0.319 | 1.07 (1.03-1.11) | <0.001 | 0.79 |
| Omega-6/FA | 0.87 (0.75-1.02) | 0.079 | 0.91 (0.86-0.96) | <0.001 | 0.718 |
| PUFA | 0.97 (0.8-1.17) | 0.721 | 0.88 (0.83-0.93) | <0.001 | 0.326 |
| PUFA/MUFA | 0.85 (0.71-1.03) | 0.095 | 0.86 (0.81-0.91) | <0.001 | 0.412 |
| PUFA/FA | 0.86 (0.74-1) | 0.055 | 0.87 (0.82-0.92) | <0.001 | 0.48 |
| SFA/FA | 1.14 (0.99-1.32) | 0.075 | 1.11 (1.06-1.18) | <0.001 | 0.993 |

Models were adjusted with age, sex, race, BMI (body mass index), MET (metabolic equivalent task), TDI (Townsend Deprivation Index), smoking and drinking status, diet score, fasting time, family history of cancer, lipid-lowering drugs, insulin, and history of chronic diseases including DM (diabetes mellitus) and CVD (cardiovascular disease). NSCLC: non-small cell lung cancer; DHA: docosahexaenoic acid; DHA/FA: docosahexaenoic acid to total fatty acids percentage; LA: linoleic acid; LA/FA: linoleic acid to total fatty acids percentage; MUFA: monounsaturated fatty acids; MUFA/FA: monounsaturated fatty acids to total fatty acids percentage; Omega-3: Omega-3 fatty acids; Omega-3/FA: Omega-3 fatty acids to total fatty acids percentage; Omega-6: Omega-6 fatty acids; Omega-6/Omega-3: Omega-6 fatty acids to Omega-3 fatty acids ratio; Omega-6/FA: Omega-6 fatty acids to total fatty acids percentage; PUFA: polyunsaturated fatty acids; PUFA/MUFA: Polyunsaturated fatty acids to monounsaturated fatty acids ratio; PUFA/FA: polyunsaturated fatty acids to total fatty acids percentage; SFA: saturated fatty acids; SFA/FA: saturated fatty acids to total fatty acids percentage; FA: total fatty acids;

| Table S12. The association between circulating fatty acids and the risk of NSCLC across the history of lowing lipid drugs. | | | | | |
| --- | --- | --- | --- | --- | --- |
| **Type** | **Yes** |  | **No** |  | ***P* for interaction** |
|  | **HR (95% CI)** | ***P*** | **HR (95% CI)** | ***P*** |  |
| DHA | 0.8 (0.71-0.9) | <0.001 | 0.92 (0.86-0.99) | 0.023 | 0.123 |
| DHA/FA | 0.87 (0.78-0.97) | 0.01 | 0.92 (0.85-0.98) | 0.017 | 0.689 |
| LA | 0.87 (0.78-0.98) | 0.018 | 0.88 (0.82-0.94) | <0.001 | 0.911 |
| LA/FA | 0.9 (0.81-0.99) | 0.034 | 0.82 (0.77-0.88) | <0.001 | 0.068 |
| MUFA/FA | 1.07 (0.98-1.17) | 0.132 | 1.15 (1.07-1.23) | <0.001 | 0.125 |
| Omega-3 | 0.8 (0.71-0.9) | <0.001 | 0.94 (0.88-1.01) | 0.084 | 0.03 |
| Omega-3/FA | 0.82 (0.73-0.92) | <0.001 | 0.93 (0.86-0.99) | 0.027 | 0.157 |
| Omega-6 | 0.88 (0.79-0.98) | 0.024 | 0.9 (0.84-0.96) | 0.002 | 0.878 |
| Omega-6/Omega-3 | 1.12 (1.01-1.24) | 0.028 | 1.07 (1.03-1.11) | 0.001 | 0.46 |
| Omega-6/FA | 0.95 (0.87-1.04) | 0.283 | 0.88 (0.82-0.94) | <0.001 | 0.068 |
| PUFA | 0.85 (0.76-0.95) | 0.003 | 0.9 (0.84-0.96) | 0.002 | 0.488 |
| PUFA/MUFA | 0.88 (0.8-0.98) | 0.017 | 0.85 (0.79-0.91) | <0.001 | 0.272 |
| PUFA/FA | 0.9 (0.83-0.99) | 0.025 | 0.85 (0.8-0.91) | <0.001 | 0.144 |
| SFA/FA | 1.1 (1.01-1.2) | 0.028 | 1.12 (1.06-1.19) | <0.001 | 0.519 |

Models were adjusted with age, sex, race, BMI (body mass index), MET (metabolic equivalent task), TDI (Townsend Deprivation Index), smoking and drinking status, diet score, fasting time, family history of cancer, lipid-lowering drugs, insulin, and history of chronic diseases including DM (diabetes mellitus) and CVD (cardiovascular disease). NSCLC: non-small cell lung cancer; DHA: docosahexaenoic acid; DHA/FA: docosahexaenoic acid to total fatty acids percentage; LA: linoleic acid; LA/FA: linoleic acid to total fatty acids percentage; MUFA: monounsaturated fatty acids; MUFA/FA: monounsaturated fatty acids to total fatty acids percentage; Omega-3: Omega-3 fatty acids; Omega-3/FA: Omega-3 fatty acids to total fatty acids percentage; Omega-6: Omega-6 fatty acids; Omega-6/Omega-3: Omega-6 fatty acids to Omega-3 fatty acids ratio; Omega-6/FA: Omega-6 fatty acids to total fatty acids percentage; PUFA: polyunsaturated fatty acids; PUFA/MUFA: Polyunsaturated fatty acids to monounsaturated fatty acids ratio; PUFA/FA: polyunsaturated fatty acids to total fatty acids percentage; SFA: saturated fatty acids; SFA/FA: saturated fatty acids to total fatty acids percentage; FA: total fatty acids;

| Table S13. The association between circulating fatty acids and the risk of NSCLC across the history of CVD. | | | | | |
| --- | --- | --- | --- | --- | --- |
| **Type** | **Yes** |  | **No** |  | ***P* for interaction** |
|  | **HR (95% CI)** | ***P*** | **HR (95% CI)** | ***P*** |  |
| DHA | 0.84 (0.71-0.99) | 0.039 | 0.9 (0.84-0.96) | 0.001 | 0.576 |
| DHA/FA | 0.86 (0.74-1) | 0.05 | 0.91 (0.85-0.97) | 0.004 | 0.542 |
| LA | 0.98 (0.84-1.13) | 0.753 | 0.86 (0.81-0.91) | <0.001 | 0.059 |
| LA/FA | 0.97 (0.85-1.12) | 0.703 | 0.82 (0.77-0.87) | <0.001 | 0.021 |
| MUFA/FA | 1.06 (0.94-1.2) | 0.353 | 1.13 (1.07-1.21) | <0.001 | 0.325 |
| Omega-3 | 0.82 (0.7-0.97) | 0.019 | 0.92 (0.86-0.98) | 0.006 | 0.291 |
| Omega-3/FA | 0.8 (0.68-0.94) | 0.007 | 0.91 (0.86-0.97) | 0.005 | 0.163 |
| Omega-6 | 0.97 (0.84-1.13) | 0.716 | 0.88 (0.83-0.94) | <0.001 | 0.108 |
| Omega-6/Omega-3 | 1.1 (1.02-1.18) | 0.011 | 1.07 (1.03-1.11) | 0.002 | 0.601 |
| Omega-6/FA | 0.98 (0.87-1.11) | 0.79 | 0.88 (0.82-0.94) | <0.001 | 0.118 |
| PUFA | 0.93 (0.81-1.08) | 0.351 | 0.88 (0.83-0.93) | <0.001 | 0.254 |
| PUFA/MUFA | 0.89 (0.77-1.02) | 0.099 | 0.85 (0.8-0.91) | <0.001 | 0.54 |
| PUFA/FA | 0.93 (0.82-1.05) | 0.23 | 0.86 (0.81-0.91) | <0.001 | 0.23 |
| SFA/FA | 1.06 (0.94-1.2) | 0.309 | 1.13 (1.07-1.19) | <0.001 | 0.368 |

Models were adjusted with age, sex, race, BMI (body mass index), MET (metabolic equivalent task), TDI (Townsend Deprivation Index), smoking and drinking status, diet score, fasting time, family history of cancer, lipid-lowering drugs, insulin, and history of chronic diseases including DM (diabetes mellitus) and CVD (cardiovascular disease). NSCLC: non-small cell lung cancer; DHA: docosahexaenoic acid; DHA/FA: docosahexaenoic acid to total fatty acids percentage; LA: linoleic acid; LA/FA: linoleic acid to total fatty acids percentage; MUFA: monounsaturated fatty acids; MUFA/FA: monounsaturated fatty acids to total fatty acids percentage; Omega-3: Omega-3 fatty acids; Omega-3/FA: Omega-3 fatty acids to total fatty acids percentage; Omega-6: Omega-6 fatty acids; Omega-6/Omega-3: Omega-6 fatty acids to Omega-3 fatty acids ratio; Omega-6/FA: Omega-6 fatty acids to total fatty acids percentage; PUFA: polyunsaturated fatty acids; PUFA/MUFA: Polyunsaturated fatty acids to monounsaturated fatty acids ratio; PUFA/FA: polyunsaturated fatty acids to total fatty acids percentage; SFA: saturated fatty acids; SFA/FA: saturated fatty acids to total fatty acids percentage; FA: total fatty acids;

| Table S14. The association between circulating fatty acids and the risk of NSCLC across drinking status. | | | | | | | |
| --- | --- | --- | --- | --- | --- | --- | --- |
| **Type** | **Never** | | **Previous** | | **Current** | | ***P* for interaction** |
|  | **HR (95% CI)** | ***P*** | **HR (95% CI)** | ***P*** | **HR (95% CI)** | ***P*** |  |
| DHA | 0.99 (0.71-1.4) | 0.968 | 1.07 (0.87-1.32) | 0.524 | 0.88 (0.82-0.93) | <0.001 | 0.116 |
| DHA/FA | 1.2 (0.89-1.61) | 0.244 | 1.13 (0.94-1.36) | 0.202 | 0.87 (0.82-0.93) | <0.001 | 0.006 |
| LA | 0.83 (0.62-1.12) | 0.226 | 0.72 (0.57-0.9) | 0.005 | 0.89 (0.84-0.95) | <0.001 | 0.106 |
| LA/FA | 0.91 (0.68-1.22) | 0.524 | 0.76 (0.62-0.93) | 0.007 | 0.84 (0.8-0.9) | <0.001 | 0.144 |
| MUFA/FA | 0.86 (0.63-1.18) | 0.356 | 1.05 (0.85-1.29) | 0.677 | 1.14 (1.07-1.2) | <0.001 | 0.13 |
| Omega-3 | 0.87 (0.62-1.22) | 0.413 | 1 (0.81-1.23) | 0.981 | 0.9 (0.85-0.96) | <0.001 | 0.344 |
| Omega-3/FA | 1.03 (0.75-1.43) | 0.834 | 1.06 (0.88-1.29) | 0.543 | 0.88 (0.82-0.93) | <0.001 | 0.06 |
| Omega-6 | 0.88 (0.66-1.18) | 0.393 | 0.75 (0.6-0.93) | 0.01 | 0.91 (0.85-0.96) | 0.002 | 0.179 |
| Omega-6/Omega-3 | 1.12 (1.06-1.19) | <0.001 | 1.12 (0.98-1.28) | 0.087 | 1.06 (1.01-1.11) | 0.01 | 0.46 |
| Omega-6/FA | 1.08 (0.8-1.45) | 0.63 | 0.88 (0.73-1.06) | 0.188 | 0.9 (0.85-0.95) | <0.001 | 0.172 |
| PUFA | 0.87 (0.64-1.17) | 0.346 | 0.78 (0.62-0.97) | 0.028 | 0.89 (0.84-0.95) | <0.001 | 0.449 |
| PUFA/MUFA | 1.08 (0.79-1.49) | 0.625 | 0.93 (0.75-1.17) | 0.542 | 0.85 (0.79-0.9) | <0.001 | 0.158 |
| PUFA/FA | 1.09 (0.8-1.48) | 0.581 | 0.9 (0.74-1.1) | 0.296 | 0.86 (0.81-0.91) | <0.001 | 0.143 |
| SFA/FA | 1.02 (0.78-1.34) | 0.888 | 1.14 (0.95-1.37) | 0.169 | 1.12 (1.06-1.18) | <0.001 | 0.519 |

Models were adjusted with age, sex, race, BMI (body mass index), MET (metabolic equivalent task), TDI (Townsend Deprivation Index), smoking and drinking status, diet score, fasting time, family history of cancer, lipid-lowering drugs, insulin, and history of chronic diseases including DM (diabetes mellitus) and CVD (cardiovascular disease). NSCLC: non-small cell lung cancer; DHA: docosahexaenoic acid; DHA/FA: docosahexaenoic acid to total fatty acids percentage; LA: linoleic acid; LA/FA: linoleic acid to total fatty acids percentage; MUFA: monounsaturated fatty acids; MUFA/FA: monounsaturated fatty acids to total fatty acids percentage; Omega-3: Omega-3 fatty acids; Omega-3/FA: Omega-3 fatty acids to total fatty acids percentage; Omega-6: Omega-6 fatty acids; Omega-6/Omega-3: Omega-6 fatty acids to Omega-3 fatty acids ratio; Omega-6/FA: Omega-6 fatty acids to total fatty acids percentage; PUFA: polyunsaturated fatty acids; PUFA/MUFA: Polyunsaturated fatty acids to monounsaturated fatty acids ratio; PUFA/FA: polyunsaturated fatty acids to total fatty acids percentage; SFA: saturated fatty acids; SFA/FA: saturated fatty acids to total fatty acids percentage; FA: total fatty acids;

| Table S15. The association between circulating fatty acids and the risk of NSCLC across smoking status. | | | | | | | |
| --- | --- | --- | --- | --- | --- | --- | --- |
| **Type** | **Never** | | **Previous** | | **Current** | | ***P* for interaction** |
|  | **HR (95% CI)** | ***P*** | **HR (95% CI)** | ***P*** | **HR (95% CI)** | ***P*** |  |
| DHA | 0.89 (0.73-1.07) | 0.223 | 0.95 (0.88-1.02) | 0.177 | 0.81 (0.73-0.9) | <0.001 | 0.184 |
| DHA/FA | 1 (0.83-1.19) | 0.963 | 0.9 (0.83-0.98) | 0.014 | 0.88 (0.79-0.97) | 0.011 | 0.271 |
| LA | 0.82 (0.68-1) | 0.048 | 0.91 (0.84-0.98) | 0.018 | 0.86 (0.78-0.94) | <0.001 | 0.713 |
| LA/FA | 1.07 (0.88-1.31) | 0.5 | 0.75 (0.69-0.81) | 0.92 | 0.85 (1.01-0.071) | <0.001 | <0.001 |
| MUFA/FA | 0.89 (0.71-1.1) | 0.271 | 1.21 (1.12-1.31) | <0.001 | 1.07 (0.98-1.16) | 0.125 | <0.001 |
| Omega-3 | 0.84 (0.69-1.02) | 0.079 | 0.99 (0.92-1.06) | 0.727 | 0.81 (0.73-0.9) | <0.001 | 0.009 |
| Omega-3/FA | 0.92 (0.77-1.11) | 0.398 | 0.94 (0.87-1.02) | 0.129 | 0.83 (0.75-0.92) | <0.001 | 0.195 |
| Omega-6 | 0.81 (0.67-0.99) | 0.035 | 0.95 (0.87-1.03) | 0.183 | 0.86 (0.79-0.94) | 0.001 | 0.467 |
| Omega-6/Omega-3 | 1.11 (1.07-1.17) | <0.001 | 1.02 (0.95-1.1) | 0.555 | 1.08 (1.02-1.15) | 0.015 | 0.118 |
| Omega-6/FA | 1.17 (0.95-1.45) | 0.144 | 0.81 (0.75-0.87) | <0.001 | 0.99 (0.91-1.08) | 0.822 | <0.001 |
| PUFA | 0.8 (0.66-0.97) | 0.022 | 0.95 (0.88-1.03) | 0.223 | 0.84 (0.76-0.92) | <0.001 | 0.201 |
| PUFA/MUFA | 1.11 (0.91-1.35) | 0.312 | 0.79 (0.72-0.85) | <0.001 | 0.91 (0.83-1) | 0.055 | <0.001 |
| PUFA/FA | 1.12 (0.9-1.38) | 0.313 | 0.79 (0.73-0.85) | <0.001 | 0.94 (0.86-1.02) | 0.134 | <0.001 |
| SFA/FA | 0.96 (0.79-1.16) | 0.667 | 1.22 (1.13-1.3) | <0.001 | 1.03 (0.95-1.11) | 0.47 | <0.001 |

Models were adjusted with age, sex, race, BMI (body mass index), MET (metabolic equivalent task), TDI (Townsend Deprivation Index), smoking and drinking status, diet score, fasting time, family history of cancer, lipid-lowering drugs, insulin, and history of chronic diseases including DM (diabetes mellitus) and CVD (cardiovascular disease). NSCLC: non-small cell lung cancer; DHA: docosahexaenoic acid; DHA/FA: docosahexaenoic acid to total fatty acids percentage; LA: linoleic acid; LA/FA: linoleic acid to total fatty acids percentage; MUFA: monounsaturated fatty acids; MUFA/FA: monounsaturated fatty acids to total fatty acids percentage; Omega-3: Omega-3 fatty acids; Omega-3/FA: Omega-3 fatty acids to total fatty acids percentage; Omega-6: Omega-6 fatty acids; Omega-6/Omega-3: Omega-6 fatty acids to Omega-3 fatty acids ratio; Omega-6/FA: Omega-6 fatty acids to total fatty acids percentage; PUFA: polyunsaturated fatty acids; PUFA/MUFA: Polyunsaturated fatty acids to monounsaturated fatty acids ratio; PUFA/FA: polyunsaturated fatty acids to total fatty acids percentage; SFA: saturated fatty acids; SFA/FA: saturated fatty acids to total fatty acids percentage; FA: total fatty acids;

**Appendix 1.** Proof of Language Editing


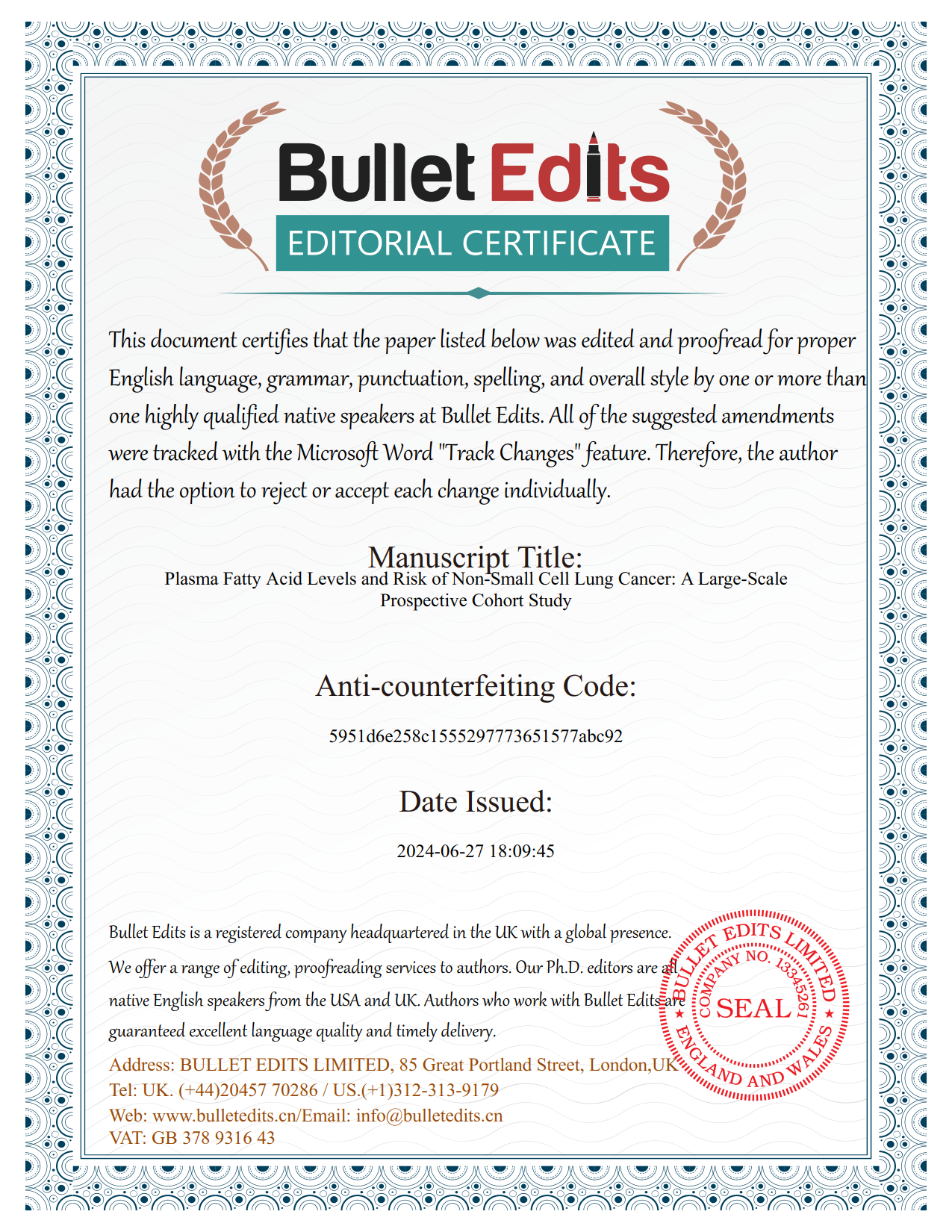

Supplement: Supplementary file 1 [file Table_1.DOCX]
